# Five computational developability guidelines for therapeutic antibody profiling

**DOI:** 10.1073/pnas.1810576116

**Published:** 2019-02-14

**Authors:** Matthew I. J. Raybould, Claire Marks, Konrad Krawczyk, Bruck Taddese, Jaroslaw Nowak, Alan P. Lewis, Alexander Bujotzek, Jiye Shi, Charlotte M. Deane

**Affiliations:** ^a^Department of Statistics, University of Oxford, Oxford OX1 3LB, United Kingdom;; ^b^Department of Antibody Discovery and Protein Engineering, MedImmune, Cambridge CB21 6GH, United Kingdom;; ^c^Computational and Modelling Sciences, GlaxoSmithKline Research and Development, Stevenage SG1 2NY, United Kingdom;; ^d^Roche Pharma Research and Early Development, Large Molecule Research, Roche Innovation Center Munich, DE-82377 Penzberg, Germany;; ^e^Chemistry Department, UCB Pharma, Slough SL1 3WE, United Kingdom

**Keywords:** therapeutic monoclonal antibodies, developability guidelines, immunoglobulin gene sequencing, surface hydrophobicity, surface charge

## Abstract

Therapeutic mAbs must not only bind to their target but must also be free from “developability issues” such as poor stability or high levels of aggregation. While small-molecule drug discovery benefits from Lipinski’s rule of five to guide the selection of molecules with appropriate biophysical properties, there is currently no in silico analog for antibody design. Here, we model the variable domain structures of a large set of post-phase-I clinical-stage antibody therapeutics (CSTs) and calculate in silico metrics to estimate their typical properties. In each case, we contextualize the CST distribution against a snapshot of the human antibody gene repertoire. We describe guideline values for five metrics thought to be implicated in poor developability: the total length of the complementarity-determining regions (CDRs), the extent and magnitude of surface hydrophobicity, positive charge and negative charge in the CDRs, and asymmetry in the net heavy- and light-chain surface charges. The guideline cutoffs for each property were derived from the values seen in CSTs, and a flagging system is proposed to identify nonconforming candidates. On two mAb drug discovery sets, we were able to selectively highlight sequences with developability issues. We make available the Therapeutic Antibody Profiler (TAP), a computational tool that builds downloadable homology models of variable domain sequences, tests them against our five developability guidelines, and reports potential sequence liabilities and canonical forms. TAP is freely available at opig.stats.ox.ac.uk/webapps/sabdab-sabpred/TAP.php.

## Significance

Immunogenicity, instability, self-association, high viscosity, polyspecificity, or poor expression can all preclude an antibody from becoming a therapeutic. Early identification of these negative characteristics is essential. Akin to the Lipinski guidelines, which measure druglikeness in small molecules, our Therapeutic Antibody Profiler highlights antibodies that possess characteristics that are rare/unseen in clinical-stage mAb therapeutics. The only required input is the variable domain sequence. We show examples where our approach would have advised against manufacturing antibodies that were found to aggregate or have poor expression.

Monoclonal antibodies (mAbs) are increasingly used as therapeutics targeting a wide range of membrane-bound or soluble antigens; of the 73 antibody therapies approved by the European Medicines Agency or Food and Drug Administration since 1986 (valid as of June 12, 2018), 10 were first approved in 2017 (1). There are many barriers to therapeutic mAb development, besides achieving the desired affinity to the antigen. These include intrinsic immunogenicity, chemical and conformational instability, self-association, high viscosity, polyspecificity, and poor expression. In vitro screening for these negative characteristics is now routine in industrial pipelines (2).

While some cases of poor developability are subtle in origin, others are less ambiguous. High levels of hydrophobicity, particularly in the highly variable complementarity-determining regions (CDRs), have repeatedly been implicated in aggregation, viscosity, and polyspecificity (2–8). Asymmetry in the net charge of the heavy- and light-chain variable domains is also correlated with self-association and viscosity at high concentrations (4, 9). Patches of positive (10) and negative (11) charge in the CDRs are linked to high rates of clearance and poor expression levels. Product heterogeneity (e.g., through oxidation, isomerization, or glycosylation) often results from specific sequence motifs liable to post- or cotranslational modification.

An improved understanding of the factors governing these biophysical properties has enabled the development of in silico assays, which are faster and cheaper than their experimental equivalents. Computational tools already facilitate the identification of sequence liabilities, for example sites of lysine glycation (12), aspartate isomerization (13), asparagine deamidation (13), and the presence of cysteines or N-linked glycosylation sites (14). A primary focus in recent years has been on designing software that can better predict aggregation proclivity. Many algorithms designed for this purpose use only the antibody sequence (4, 7, 8), although some suggest an analogous equation to use if a structure is available (4). One purely structure-based method is the Structural Aggregation Propensity (SAP) metric (5), later included in the Developability Index (6). This has been shown to detect aggregation-prone regions, such as surface patches (15), and to be able to rank candidates relative to a known antibody developability profile (11), using a closely related antibody crystal structure. It is likely that SAP’s atomic-resolution analysis would be too sensitive to use in comparing static homology models of diverse antibodies, given the current accuracy of structure prediction (16).

An alternative approach to predict antibodies likely to have poor developability profiles is to highlight those candidates whose characteristics differ greatly from clinically tested therapeutic mAbs; a similar strategy in the field of pharmacokinetics led to the Lipinski rules for small-molecule drug design (17). Here, we build 3D models of a large set of post-phase-I therapeutics and survey their sequence and structural properties. These values are then contextualized against human immunoglobulin gene sequencing (Ig-seq) sequences and models, to see where therapeutics share and deviate from the properties of human mAbs.

Using the distributions of these properties, we build the Therapeutic Antibody Profiler (TAP), a computational tool that highlights antibodies with anomalous values compared with therapeutics. TAP builds a downloadable structural model of an antibody variable domain sequence and tests it against guideline thresholds of five calculated measures likely to be linked to poor developability. It also reports potential sequence liabilities and all non-CDRH3 loop canonical forms.

## Results

### Sequence Data.

As a dataset of mAbs unlikely to suffer with developability issues, we used the variable domain heavy- and light-chain sequences of 137 clinical-stage antibody therapeutics (137 CSTs) (18). To contextualize the properties of the CST set, we retrieved Vander Heiden’s recent snapshot of the human antibody repertoire from the Observed Antibody Space database (19, 20) (human VdH Ig-seq). We also used a larger proprietary dataset procured by UCB Pharma Ltd. (human UCB Ig-seq). All comparisons in the paper are made to the Vander Heiden data, with UCB comparisons available in *SI Appendix*. Each human Ig-seq dataset was analyzed as a set of nonredundant heavy or light chains (human Ig-seq nonredundant chains) and as a set of nonredundant CDR sequences (human Ig-seq nonredundant CDRs). We chose these Ig-seq datasets as they contain simultaneously sequenced heavy and light chains and so are a promising starting point for realistic in silico pairing, required to make complete structural models.

### Model Structures.

High-quality structural information is critical to accurately predict the surface properties of antibodies. As crystal structures are often unavailable, or difficult to attain, accurate modeling is a necessary step of an effective antibody profiler. Accordingly, all our comparisons are made between models, even when crystal structures are available, to avoid a bias in terms of structural quality [modeling introduces a systematic bias toward higher values for our patches of surface hydrophobicity (PSH) metric; see *SI Appendix*, Figs. S9 and S10]. ABodyBuilder (21) was run on the 56 CSTs with a reference Protein Data Bank (PDB) (22) structure (as of May 4, 2018). Sequence-identical templates were not included, and each resulting model was aligned to its reference to evaluate the backbone rmsd across all IMGT (international ImMunoGeneTics information system) regions (*SI Appendix*, *Methods*). The mean framework and CDR rmsds (*SI Appendix*, Table S1) were commensurate with the current state of the art (16). For our structural property calculations, we class surface-exposed residues as having a side chain with relative accessible surface area (ASA_rel,X_) ≥7.5%, compared with alanine-X-alanine for each residue X (23, 24). Using this definition, we identified all exposed residues in the models and PDB structures. Of the 7,057 exposed crystal structure residues, only 265 (3.76%) were wrongly assigned as buried in the models.

As these results suggest that ABodyBuilder models are accurate enough for our analysis, we used this software to model all 137 CSTs (137 CST models) and diverse subsets of paired human VdH Ig-seq chains (14,072 human VdH Ig-seq models) and paired human UCB Ig-seq chains (19,019 human UCB Ig-seq models). The pairing and modeling protocol was designed to capture the sequence and structural diversity in each dataset, within the constraints of modelability and computational expense (*SI Appendix*, *Methods*). We then performed a series of in silico assays to determine the TAP metrics.

### IMGT CDR Lengths.

Loop length has a major impact on the nature of antigen binding. For example, if an antibody has a long CDRH3 loop, it tends to form most of the interactions with an antigen, while shorter CDRH3 loops contribute to concave binding sites where other CDRs more often assist in binding (25).

**Fig. 1.**
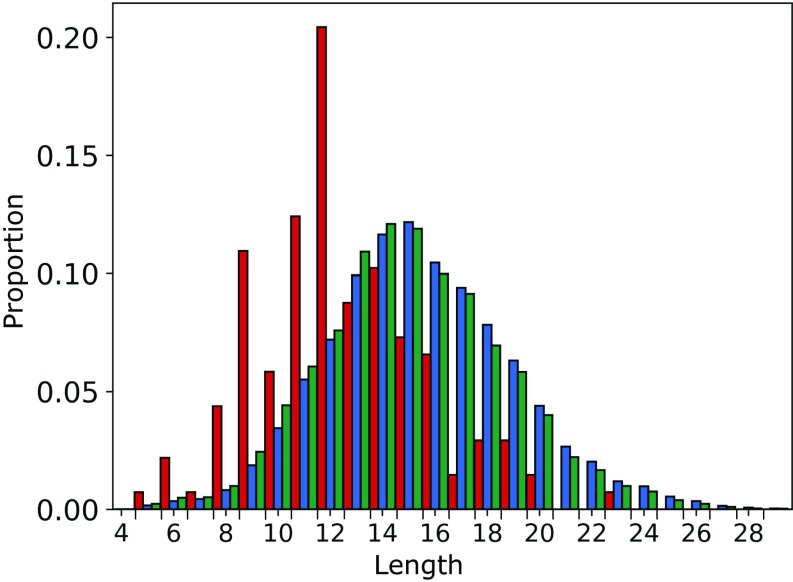
Comparing the CDRH3 length distributions of the 137 CSTs (red), 105,458 human VdH Ig-seq nonredundant CDRH3s (blue), and 551,193 human VdH Ig-seq nonredundant heavy chains (green). The CSTs have a lower median CDRH3 length.

The 137 CST and human Ig-seq sequences were IMGT-numbered (26), and IMGT CDR definitions were used to split the sequences by region. The 137 CST CDRH3 loops had a median length of 12, compared with 15 for the human VdH Ig-seq dataset (Fig. 1). In the case of CDRL3 the distributions were closer, with a median length of 9 for the 137 CSTs and the human VdH Ig-seq data (*SI Appendix*, Fig. S1*E*).

To test whether hybridomal development might account for these findings—as it is known that mouse antibodies tend to have shorter CDRH3 loops than human antibodies (27)—we split the 137 CST dataset by developmental origin (*SI Appendix*, Fig. S3). Fully human therapeutics were disproportionately represented at longer CDRH3s (mean: 13.21, median: 12), compared with chimeric, humanized, or fully murine therapeutics (mean: 11.91, median: 12). However, both therapeutic subsets still have shorter CDRH3s than human-expressed antibodies.

The combined length of all CDRs for each antibody in the 137 CST dataset had a median value 48 (*SI Appendix*, Fig. S4). The 137 CST total CDR length was highly correlated to CDRH3 length (Pearson’s correlation coefficient of +0.77, with a two-tailed *P* value of 2.44e^−28^). While neither human Ig-seq dataset is natively paired, our artificially paired human Ig-seq models had a total CDR length distribution similar to that of the CSTs (*SI Appendix*, Fig. S4), so CDR length should not bias comparisons in other metrics. As the total length of the CDRs captures both binding-site shape (lower value and more concave) and CDRH3 length (typically shorter in CSTs than our human Ig-seq heavy chains), this metric was selected for inclusion in the final five TAP guidelines.

### Canonical Forms.

In natural antibodies, all CDR loops, apart from CDRH3, are thought to fall into structural classes known as canonical forms (28, 29). We assigned length-independent canonical forms (*Methods*) to the 137 CST and human Ig-seq models. All assignable CST model CDRs were labeled with a canonical form also present in at least one human Ig-seq model dataset (*SI Appendix*, Figs. S5 and S6). Fewer than 19% of CST CDRs remained unassigned in each loop region, suggesting that, despite engineering, a clear majority of non-CDRH3 therapeutic CDR loops adopt well-characterized canonical forms. TAP reports the canonical form of each modeled loop, highlighting if any cannot be assigned.

### Hydrophobicity.

Hydrophobicity in the CDR regions has been repeatedly linked to aggregation propensity in mAbs (2, 6–8). Using our homology models, we estimated the effective hydrophobicity of each residue by considering not only its degree of apolarity but also whether or not it is solvent-exposed [side-chain ASA_rel_ ≥7.5% (23, 24)]. As the energy of the hydrophobic effect is approximately proportional to the interface area (30), we developed a metric, PSH (*Methods*), that yields higher scores if hydrophobic residues tend to neighbor one another in a region, rather than being evenly separated. We evaluated PSH for the 137 CST and human Ig-seq models across two regions [the CDR vicinity (*Methods*) and the entire variable (Fv) region] and with five different hydrophobicity scales (31–35).

**Fig. 2.**
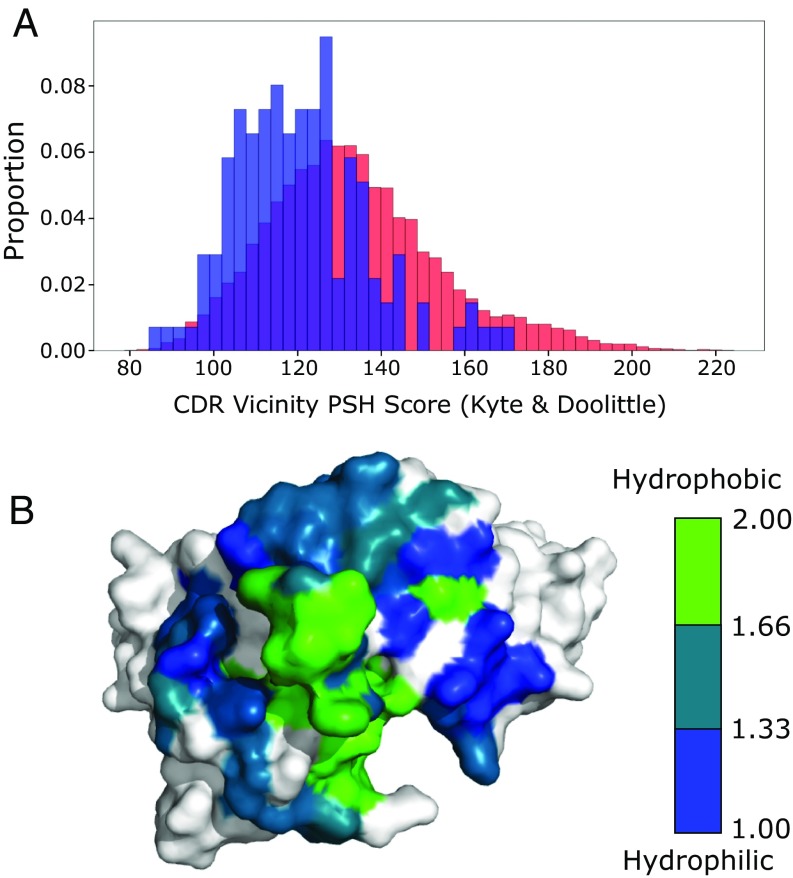
(*A*) CDR vicinity PSH scores across the 137 CST (blue) and human VdH Ig-seq (red) models. The CSTs are underrepresented at higher PSH values. (*B*) Galiximab (Kyte and Doolittle CDR vicinity PSH score of 167.89) has a large surface-exposed patch of hydrophobicity in its CDRH3 loop. Heavy-and light-chain surfaces outside the CDR vicinity are colored in white.

The results of all hydrophobicity scales were highly correlated (e.g., R^2^ ≥0.91 between all scales in the CDR vicinity), and so we use the Kyte and Doolittle (31) scale for all subsequent comparisons. The mean CDR vicinity PSH values for the CST and human VdH Ig-seq distributions were 123.30 ± 16.60 and 133.76 ± 21.08, respectively (Fig. 2*A*). CSTs were noticeably underrepresented at higher CDR PSH values; galiximab is a rare example of a therapeutic antibody with a high value (Fig. 2*B*). A similar divergence occurred across the entire Fv region, with mean values of 357.69 ± 22.95 and 370.56 ± 24.45, respectively (*SI Appendix*, Fig. S7), implying the primary difference occurs within the CDRs. This supports the theory that the high concentration conditions under which therapeutics are stored may render them less tolerant of large patches of hydrophobicity in the highly exposed CDR vicinity and also suggests that a subset of natural human antibodies would be unsuitable therapeutic candidates. We therefore included the CDR vicinity PSH score as a TAP metric.

### Charge.

Surface patches of positive or negative charge have also been linked to negative biophysical characteristics (10, 11). We calculated two metrics designed to highlight regions of dense charge: the patches of positive charge (PPC) and patches of negative charge (PNC) measures (*Methods*). All surface residues were initially assigned the appropriate charge for their averaged pK_a_ values, as neighboring residues appear to have a limited effect at pH 7.4 (4). The charge of residues found to be engaging in salt bridges was then revised to zero.

**Fig. 3.**
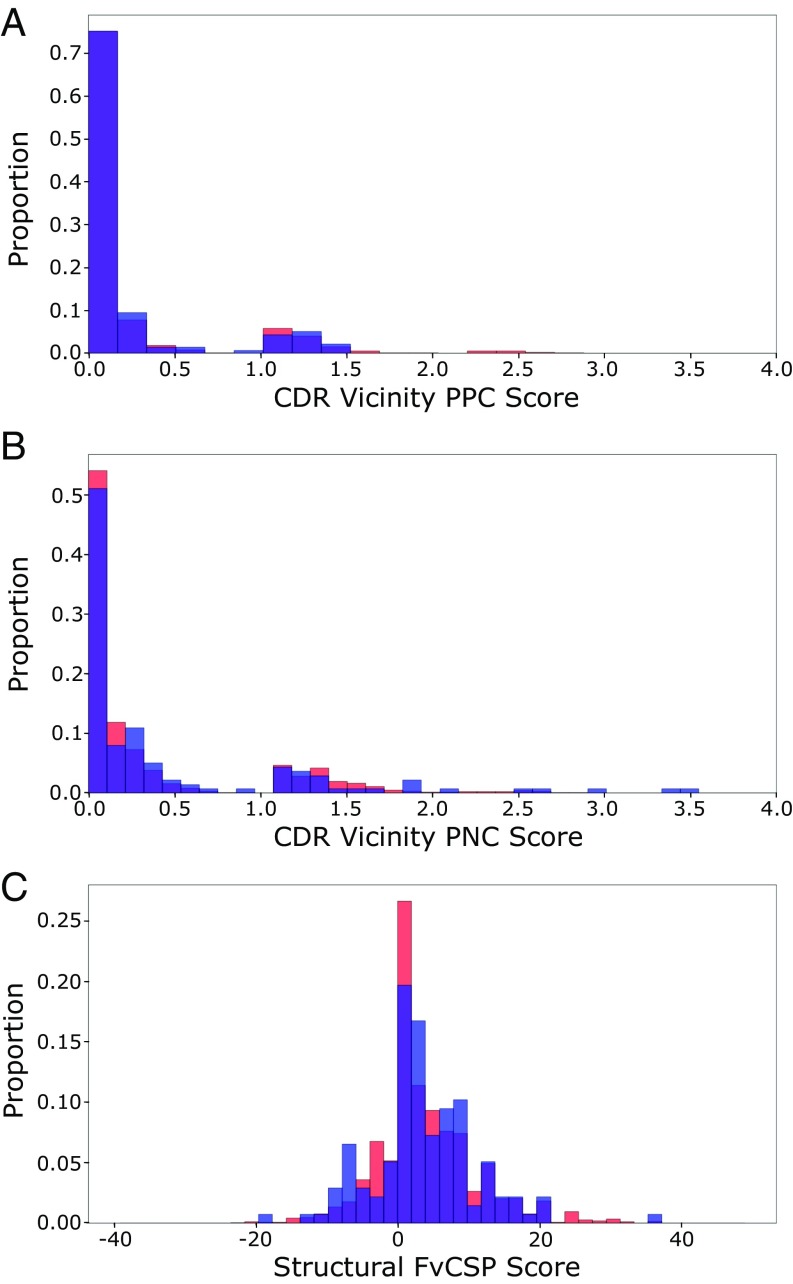
Histograms of 137 CST (blue) and human VdH Ig-seq model (red) values for the (*A*) PPC and (*B*) PNC metrics in the CDR vicinity. In both measures, the datasets are biased away from higher scores. (*C*) Histogram of structural Fv charge symmetry parameter values. Both datasets show a bias away from negative values.

The 137 CST models tend to avoid patches of charge in their CDR vicinities, with 88.32% and 80.30% having PPC (Fig. 3*A*) and PNC (Fig. 3*B*) values below 1, respectively. The human VdH Ig-seq models displayed similar PPC and PNC distributions. Both PPC and PNC assays were carried forward as TAP metrics.

When mAbs have oppositely charged V_H_ and V_L_ chains, they typically have higher in vitro viscosity values (4). This aggregate-inducing electrostatic attraction is captured at the sequence level by the Fv charge symmetry parameter (FvCSP) metric—the mAb tends to be more viscous if the product of net V_H_ and V_L_ charges is negative (4). Harnessing our structural models, we calculated a variant (the structural Fv charge symmetry parameter, SFvCSP), which only includes residues that are surface-exposed, and not locked in salt bridges, in the evaluation of net charge. In galiximab, for example, we “correct” the charge of arginine H108 and aspartic acid L56 to 0, as the model indicates that they form a salt bridge. The charges of the glutamic acid at position H6, the aspartic acids at positions H107, L98, and L108, and the histidine at position L40 are ignored as their side chains are buried. The FvCSP score for this antibody would be 0 (net heavy chain charge of 0, net light chain charge of −2.9), while the SFvCSP score is +2.0 (net heavy chain charge of +2, net light chain charge of +1). A similarly low percentage of CST models (21.9%) and human VdH Ig-seq models (20.8%) had negative SFvCSP scores (Fig. 3C), with mean values of 3.34 ± 7.44 and 3.67 ± 7.40, respectively. With such a bias away from negative products, we chose the SFvCSP as our final TAP property.

### The Importance of Modeling.

We then mined SAbDab (36) to find all of the human, nonengineered, nonredundant (at 100% sequence identity) X-ray crystal structures in the PDB (22). We found only 33 such mAbs (identities listed in Dataset S1), as most human mAb PDB entries involve some degree of engineering. Calculating their TAP metric values, we found approximately the same difference in mean CDR vicinity PSH score between therapeutic and human crystal structures as we did between therapeutic and human VdH Ig-seq models (−9.69 and −10.46 respectively; see *SI Appendix*, Table S2). However, if we had compared human structures to therapeutic models, we would not have detected a significant difference (therapeutic models: 123.30 ± 16.60; human structures: 124.61 ± 16.54). This systematic bias toward higher PSH values in models is seen most clearly when comparing the values for CST crystal structures with CST models (*SI Appendix*, Fig. S9).

### Developability Guidelines.

When comparing the TAP metric values obtained for the 56 CST structures and their corresponding models, we saw positive correlations across all metrics (*SI Appendix*, Fig. S10). This indicates that calculations performed on ABodyBuilder models are typically predictive of the results that would be obtained from a crystal structure, and therefore that threshold values derived from models are informative.

While CSTs predictably share many features in common with human antibodies, our CDR length and hydrophobicity distributions imply that not every human antibody would make a good therapeutic. Consequently, our developability guidelines were set solely by CST values across the five selected metrics (Table 1). An amber flag indicates that the antibody lies within the extremes of the distribution, whereas a red flag indicates a previously unobserved value for that property.

**Table 1.** TAP amber and red flag cut-off thresholds, with respect to the clinical-stage therapeutic distributions

Metric	Amber flag region	Red flag region
1. Total CDR length	Bottom 5%, top 5%	Above or below
2. PSH, CDR vicinity	Bottom 5%, top 5%	Above or below
3. PPC, CDR vicinity	Top 5%	Above
4. PNC, CDR vicinity	Top 5%	Above
5. SFvCSP	Bottom 5%	Below

To confirm that these threshold definitions do not typically flag mAbs without developability issues, we identified a further 105 mAb therapies (105 CSTs, listed in Dataset S2), not included in the 137 CST dataset, that had advanced to at least phase II in clinical development.

Only eight of this set (7.69%) were assigned a red developability flag according to the boundaries set by the 137 CSTs, an average of 0.08 red flags per newly tested therapeutic (*SI Appendix*, Table S3). Erenumab received the most red flags—for total CDR length (60), CDR vicinity PSH (173.85), and CDR vicinity PPC (1.53). All other red-flagged therapeutics received only one: rafivirumab for total CDR length (60); intetumumab for CDR PSH (83.84); adacanumab, derlotuximab, lanadelumab, and teprotumumab for CDR PPC (2.67, 2.66, 2.48, and 3.16, respectively); and quilizumab for Fv charge asymmetry (−20.40). The low red-flagging rate confirms that these guideline characteristics are highly conserved across therapeutic-like antibodies. Incorporating both sets of CSTs into a larger dataset (242 CSTs) led to the new guideline values shown in Table 2. While most metrics were only slightly adjusted, the PPC thresholds changed quite significantly. As a result, we performed statistical sampling over our TAP metric distributions to give a sense of the error that might be inherent in these new threshold values (*SI Appendix*, *Methods* and Table S4). All 242 CST TAP metric values are listed in Dataset S3.

**Table 2.** TAP amber and red flag regions, as defined by the entire set of 242 CSTs

Metric	Amber flag region	Red flag region
Total CDR length	54 ≤ L ≤ 60	L > 60
PSH, CDR vicinity	83.84 ≤ PSH ≤ 100.71	PSH < 83.84
	156.20 ≤ PSH ≤ 173.85	PSH > 173.85
PPC, CDR vicinity	1.25 ≤ PPC ≤ 3.16	PPC > 3.16
PNC, CDR vicinity	1.84 ≤ PNC ≤ 3.50	PNC > 3.50
SFvCSP	-20.40 ≤ SFvCSP ≤ -6.30	SFvCSP < -20.40

PSH score is calculated with the Kyte and Doolittle (31) hydrophobicity scale. L, length.

### Case Studies.

**Fig. 4.**
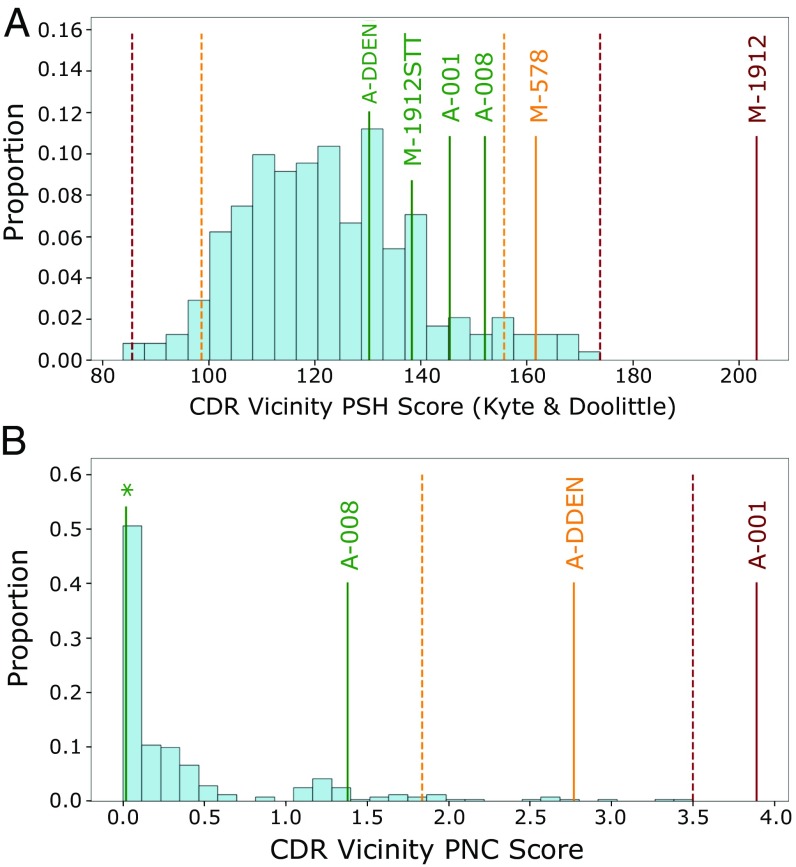
The (*A*) CDR vicinity PSH and (*B*) CDR vicinity PNC metrics for the combined set of 242 CSTs (light blue) and MedImmune case studies (colored by assigned flag). MEDI-578, MEDI-1912, and MEDI-1912STT all have the CDR vicinity PNC value labeled by an asterisk. Amber and red dashed lines delineate the 242 CST guideline thresholds. Case studies with prohibitive developability issues (MEDI-1912, AB001) are red-flagged for the PSH and PNC metrics, respectively. Engineered versions without developability issues (MEDI-1912STT, AB001DDEN) return to the range of values previously seen in CSTs for all metrics. MEDI-578/1912/1912STT are labeled as M-578/1912/1912STT, and AB-001/008/001DDEN are labeled as A-001/008/DDEN for legibility.

We tested whether these updated guideline values could highlight candidates with developability problems by building models and running TAP on two datasets supplied by MedImmune (Fig. 4). A lead anti-NGF antibody, MEDI-578, showed minor aggregation issues during in vitro testing, of a level usually rectifiable in development, whereas the affinity-matured version, MEDI-1912, exhibited unrectifiably high levels of aggregation (37). This observation was rationalized through SAP score (6) values, indicating that a large hydrophobic patch on the surface was responsible. TAP assigns MEDI-578 an amber flag and MEDI-1912 a red flag—by a large margin—in the CDR vicinity PSH metric (Fig. 4*A*). The paper describes how back-mutation of three hydrophobic residues in MEDI-1912 to those of MEDI-578 led to MEDI-1912STT, fixing the aggregation issue while maintaining potency. TAP assigns MEDI-1912STT no developability flags (Fig. 4*A*).

A lead anti-IL13 candidate, AB008, had no developability issues, but the affinity-matured version, AB001, had very poor levels of expression (seven times lower than AB008) (11). The authors highlighted the role of four consecutive negatively charged residues in the L2 loop—mutation of the fourth negatively charged residue to neutral asparagine (AB001DDEN) was able to stabilize the loop backbone, mitigating the ionic repulsion of the DDE motif, and returning acceptable levels of expression. TAP assigns no developability flags to AB008 but a red flag to AB001 and an amber flag to AB001DDEN for its CDR vicinity PNC metric (Fig. 4*B*), again red-flagging the candidate with prohibitive developability issues. Both AB001 and AB008, confirmed monomers in solution (11), did not flag for CDR vicinity PSH score (Fig. 4*A*).

### Web Application.

We have packaged the TAP into a web application, available at opig.stats.ox.ac.uk/webapps/sabdab-sabpred/TAP.php. TAP only requires the heavy- and light-chain variable domain sequences as an input, returning a detailed profile of an antibody with a typical runtime of less than 30 s. Flags (green, amber, or red) are assigned to each of the TAP metrics, with accompanying histograms. An interactive molecular viewer allows the user to visualize hydrophobicity (*SI Appendix*, Fig. S11*A*), charge, and probable sequence liabilities on the antibody model surface. Estimated model quality can be easily accessed to help guide interpretation of the results (*SI Appendix*, Fig. S11*B*). Finally, canonical forms are assigned to each non-CDRH3 loop. A full sample output is shown in *SI Appendix*, Fig. S12.

## Discussion

We have analyzed several properties linked to poor developability across 242 post-phase-I therapeutics, with the assumption that mAbs that have reached this stage of clinical trials have characteristics amenable to therapeutic development.

By analyzing these properties, we have found evidence that suggests that not every human antibody would make a good therapeutic. This would be somewhat intuitive, as therapeutics suffer a range of stresses during development (including variation in pH and temperature, sheer forces, and high-concentration storage conditions) that human-expressed antibodies are not exposed to. The TAP metrics therefore depend on the values seen across CSTs alone.

Our simple TAP guidelines will not capture the whole spectrum of developability issues. For example, they will not detect sources of immunogenicity or more subtle mechanisms that lead to poor stability. Nevertheless, we have shown that the TAP guidelines can selectively highlight antibodies with expression or aggregation issues (11, 37).

We intend to recalculate the threshold values regularly to include new mAbs that have entered phase II of clinical trials. It will also allow for the inevitable fluctuation in PSH, PPC, PNC, and SFvCSP values returned by CSTs, as ABodyBuilder models improve as the number of antibodies in the PDB increases (36).

When enough CSTs are available, it may be possible to stratify the therapeutic guidelines into subclasses. For example, separate thresholds could be considered for mAbs involving kappa or lambda light chains. Lambda light chains tend to contribute to higher average CDR vicinity PSH values across our 242 CST, 14,072 human VdH Ig-seq, and 19,019 human UCB Ig-seq models (*SI Appendix*, Table S5); DeKosky et al. (38) also found, across their 2,000 natively paired models of mAbs, that lambda CDRL3 loops are significantly more hydrophobic than their kappa equivalents. As we currently have only 25 lambda light-chain CSTs, we do not have enough data to safely determine a guideline threshold. Nevertheless, as around 90% of post-phase-I CSTs are derived from kappa light chains, this could suggest that hydrophobicity-driven developability issues are far more prevalent when using leads containing lambda light chains.

Other subclasses could include clinical trial progression, active/discontinued trial status, or therapeutic species origin. At this stage, neither splitting by clinical progression (*SI Appendix*, Table S6) nor drug campaign status (*SI Appendix*, Table S7) leads to significant differences in mean metric values. Human and humanized mAbs have noticeably higher mean PSH values than chimeric or mouse mAbs (*SI Appendix*, Table S8)—with the caveat that there are only 36 mAbs in the latter category.

As with the Lipinski rule of five, the thresholds themselves should not be interpreted as hard-and-fast rules, and the distance of red-flagged candidates outside the previously observed bounds should be taken into consideration. Advances in process development and formulation may soon redefine the limits of permissible values (18).

## Methods

All 242 CST sequences are supplied in Dataset S2, and the 551,193 heavy-and 1,359,745 light-chain nonredundant, “healthy” human VdH Ig-seq sequences can be obtained from the Observed Antibody Space database (20). The 4,587,907 heavy-chain and 7,120,000 light-chain nonredundant human UCB Ig-seq sequences are available as separated CDR and framework regions at antibodymap.org/structure. Therapeutic models and human VdH Ig-seq models can be downloaded from opig.stats.ox.ac.uk/resources. The pairing/modeling protocol used to derive the human Ig-seq model datasets can be found in *SI Appendix*, *Methods*.

### CSTs.

The initial set of 137 CST antibody sequences was sourced from the supporting information of Jain et al. (18). The test set of 105 CST sequences was found through an extensive search of online resources, including the IMGT mAb (www.imgt.org/mAb-DB/) and Antibody Society (https://www.antibodysociety.org/late-stage-clinical-pipeline/) databases. The names, sequences, and metadata for each CST are supplied in Dataset S2, with PDB structures (where available) listed at opig.stats.ox.ac.uk/webapps/sabdab-sabpred/Therapeutic.html.

### Canonical Forms.

A length-independent canonical form clustering protocol (39) was run on the North-defined (40) CDR loops of a SAbDab (36) snapshot from September 26, 2017. Model loops were inferred to have identical canonical forms to the template used by ABodyBuilder (21).

### Surface-Exposed Residues.

Residues defined as “surface-exposed” have ≥7.5% relative exposure (24) across side-chain atoms, compared with the open-chain form alanine-R-alanine, as calculated with the Shrake and Rupley algorithm (23).

### CDR Vicinity.

The “CDR vicinity” comprises every surface-exposed IMGT-defined CDR and anchor residue, and all other surface-exposed residues with a heavy atom within a 4-Å radius.

### Salt Bridges.

Salt bridges were defined as pairs of lysines/arginines and aspartic acids/glutamic acids with a N^+^−O^−^ distance ≤3.2 Å.

### Hydrophobicity.

Where R_1_ and R_2_ are two surface-exposed residues with a closest heavy-atom distance, r_12_, <7.5 Å and H(R,S) is the normalized hydrophobicity score (between 1 and 2) for residue R in scheme S, the PSH metric can be calculated as $\sum_{R_{1}R_{2}}\frac{H\left(R_{1},S\right)H\left(R_{2},S\right)}{r_{12}^{2}}$. The hydrophobicity scales tested were Kyte and Doolittle (31), Wimley and White (32), Hessa et al. (33), Eisenberg and McLachlan (34), and Black and Mould (35). Salt-bridge residues were assigned the same value as glycine in each hydrophobicity scale.

### Charge.

The following charges were assigned by sequence: aspartic acid, −1; glutamic acid, −1; lysine, +1; arginine, +1; and histidine, +0.1 (Henderson–Hasselbalch equation applied: pK_a_ 6, pH 7.4, and rounded up to one decimal place). Tyrosine hydroxyl deprotonation was not considered. Salt-bridge residues were assigned a charge of 0. The PPC and PNC metrics are analogous in form to PSH, with H(R,S) substituted for |Q(R)|, the absolute value of the charge assigned to residue R. SFvCSP values were calculated as $\left[\sum_{R_{H}}Q\left(R_{H}\right)\right]\left[\sum_{R_{L}}Q\left(R_{L}\right)\right]$, where R_H_ and R_L_ are surface-exposed V_H_ and V_L_ residues, respectively.

## Supplementary Material

Supplementary File

Supplementary File

Supplementary File

Supplementary File
