# Supplementary material for: Five computational developability guidelines for therapeutic antibody profiling
Source: Proc Natl Acad Sci U S A. 2019 Feb 14;116(10):4025–30. doi: 10.1073/pnas.1810576116 (PMC6410772; doi:10.1073/pnas.1810576116)
Supplement: Supplementary File [file pnas.1810576116.sapp.pdf]

PNAS
www.pnas.org

Supplementary Information for

The Therapeutic Antibody Profiler (TAP): Five Computational Developability Guidelines

Raybould MIJ *et al.*

Corresponding Author: Charlotte M. Deane
E-mail: deane@stats.ox.ac.uk

This PDF file includes:

Supplementary text
Figs. S1 to S12
Tables S1 to S8
Captions for Databases S1 to S3
References for SI reference citations

Other supplementary materials for this manuscript include the following:

Databases S1 to S3

www.pnas.org/cgi/doi/10.1073/pnas.1810576116

Supporting Information Text

Methods

Human VdH Ig-seq Sequencing Techniques. The sequencing procedure followed to obtain the human VdH Ig-seq nucleotide reads is described in the manuscript and SI of Vander Heiden *et al.* (1).

Human UCB Ig-seq Sequencing Techniques. 1. Commercially Sourced RNA Samples. Three different total RNA samples at 1µg/µl were sourced from Clontech Laboratories comprising 50µg prepared from normal human spleen (SP), pooled from 12 male/female Caucasians aged between 18 and 54 years, 10µg from normal bone marrow (BM) from 56 Asian males aged between 22 and 85 and 10µg from normal human peripheral leukocytes (PBL) from 426 male/female Asians aged between 18 and 54 (PBL).

2. Reverse Transcription and C-region Specific Oligonucleotides. Multiple reverse transcription (RT) reactions were done for each RNA source (SP: 12xVH, 6xVK, 6xVL BM: 6xVH, 2xVK, 2xVL PBL: 6xVH, 2xVK, 2xVL) using human antibody constant region reverse oligonucleotides specific for the 3' ends of the CH1 gene of IgM and the 3' ends of the C-kappa and C-lambda genes. After an initial denaturation at 65°C for 5 min in the presence of oligonucleotide and dNTPs multiple 20µl reactions each containing 1ug RNA, 200U Superscript III (Life Technologies), 20U RNasin (Promega), 5mM DTT, 500µM dNTPs and 1µM oligonucleotide were incubated for 60 min at 50°C and 15 min at 70°C before being frozen at -20°C.

3. Primary PCRs. Twelve family-restricted primary PCRs (5xVH, 4xVK, 3xVL families) were done on each of the three cDNA template samples SP, BM and PBL. Where a V-gene family required more than one oligonucleotide these were mixed in proportions equivalent to estimates of sequence frequency. A total of 304 individual (192xVH, 64xVK and 48xVL) 25μl buffered reactions, each with 1μl of cDNA as template, consisted of 1mM dNTPs, 1.5mM MgSO4, 4μM forward and 4μM reverse oligonucleotides and 0.5U KOD hot start DNA polymerase (Merck Millipore). After an initial denaturation step of 96°C for 2 min, PCR cycling conditions for all reactions were 96°C for 15s, 55°C for 15s, 72°C for 15s for 40 cycles followed by a final extension step for 5 min at 72°C.

4. Secondary PCRs. An equivalent 304 (192xVH, 64xVK, 48xVL) 50μl individual secondary PCRs were done, keeping the DNA samples from the primary reactions separate in order to maximize V-gene diversity. The reactions, each with 2μl of primary PCR as template, had matched components and cycling conditions to the primary reactions except the cycle number was reduced to 30. Once again V-region family oligonucleotide sets were kept separate and members within each family were mixed at the pre-determined proportions.

5. Sample Preparation for the Oxford Sequencing Centre The secondary PCR products for each of the specific V-gene family (VH1-6, VK1-4, VL1-2) were pooled, giving 12 samples. Approximately 1μg from each pool was analysed by agarose gel electrophoresis (Invitrogen UltraPureTM agarose) and the DNA of approximately 400bp was excised, gel extracted (Qiagen) and eluted into 50μl of water at a final concentrations of between 10-75ng/μl to be analysed by paired-end next generation sequencing on an Illumina MiSeq machine at the Oxford Genomics Centre (OGC) at the Wellcome Trust Centre for Human Genetics, Oxford.

Bioinformatic Annotation of Ig-seq Sequences. IgBLAST 1.4.0 (2) was used with Human V, D & J germline reference sets from IMGT for both heavy and light chains to germline annotate the full length reads. A custom Java pipeline was used to process the IgBLAST output and identify high quality sequences. The criteria for this were as follows: identified germline V & J genes; full length variable chain sequence (1-2 bp missing at 5' & 3' end was permitted); absence of stop codons or ambiguous nucleotide calls. Sequences successfully extracted were saved into flat files together with the identifiers of their assigned germline sequences. This Ig-seq dataset was translated, then IMGT-numbered and filtered by ANARCI to remove poor-quality reads (3). This parsing removes sequences that do not align, have IMGT CDRH3 lengths $\geq$ 37, possess indels in the canonical CDRs or framework regions, start at IMGT position 24 or later, or have a J gene with sequence identity less than 50% to known IMGT germlines. It is at this stage that all Ig-seq datasets in the Observed Antibody Space database are supplied to researchers (4).

Preparation of the Human VdH Ig-seq Dataset for Analysis. For structural comparisons, where heavy and light chains must be paired and then modeled, computational expense required us to reduce the size of the dataset, while still retaining as true an indication as possible of the sequence and structural variation inherent within the immunoglobin repertoire. The chains were therefore filtered and paired according to the following protocol, optimized for computational efficiency. The ANARCI-filtered dataset was trimmed to remove heavy and light chain sequences that contained IMGT CDRs (5) (CDRH1-2, CDRL1-3) for which SCALOP (6) could not predict a canonical form (inability to predict a canonical form is highly linked to an inability to model the sequence). Then FREAD (7–9) was run on each surviving heavy chain's CDRH3 loop to remove chains for which no viable CDRH3 template can be found. On this reduced number of heavy and light chains, the full version of FREAD was then run to assign templates to all loops. The V_H and V_L sequences were then greedily clustered with a 90% sequence identity threshold, and with a restriction that each cluster must only contain sequences of identical length. Clusters with fewer than 10 members were discarded to remove erroneous reads. From the surviving clusters, only the sequence with the lowest median sequence identity to the rest of the cluster members was retained. The chains were then paired by assigning the V_H-V_L angle from a diverse set of 989 complete antibodies from SAbDab (10) (selected in May 2016) to the heavy-light chain pair, if the sequence identity across the heavy-light chain interface residues exceeds 0.82 (11). If multiple viable templates exist, the one with highest sequence identity was chosen. Low-resolution structural clustering of CDR binding sites was then performed

in a two-step process. If the orientation RMSD between the orientation template of the newly-considered antibody and the templates of all other previously-considered antibodies exceeds 1.5 Å, the binding site was classified as distinct based on V_H-V_L angle alone, and the antibody was retained. Otherwise, CDR distance was evaluated as:

$$\sqrt{\frac{\sum_{X}^{(L1-L3,H1-H3)} DTW^2_{CDR-X} max(L_{CDR-X,1}, L_{CDR-X,2})}{\sum_{X}^{(L1-L3,H1-H3)} max(L_{CDR-X,1}, L_{CDR-X,2})}}$$

where the summation is over all CDRs, $DTW_{CDR\text{-}X}$ is the Dynamic Time Warping (12) distance between CDR templates, and $max(L_{CDR\text{-}X,1}, L_{CDR\text{-}X,2})$ is the maximum length of the two loops. The new antibody was retained if this equation returned a value greater than 1.0 Å to all other previously-considered antibodies. The surviving Fvs were then homology modeled using ABodyBuilder (11), and these 14,072 models became the Human VdH Ig-seq models dataset. The resulting total CDR length distribution of this set of models proved to be similar to that of the set of 137 CSTs (Fig. S4A).

Preparation of the Human UCB Ig-seq Dataset for Analysis. The UCB dataset was prepared in an analogous manner to the VdH Ig-seq dataset, but with a few modifications. As it was prepared before SCALOP (6) had been developed, FREAD was run on all chains at the beginning to remove chains without three Chothia CDR loop templates. Being far larger than the VdH Ig-seq dataset (about 10x the number of heavy and light chain sequences), around 66,000 models were obtained after DTW clustering and ABodyBuilder modeling. To reduce this number further, higher-resolution structural clustering was performed on the CDR coordinates. The RMSD between common CDR residues in each pair of models, after Kabsch alignment (13), was evaluated and stored in a matrix, which was clustered using the Unweighted Pair Group Method with Arithmetic Mean (UPGMA) hierarchical method (14), with a 1.0 Å cutoff. A representative of each cluster was selected and the resulting 19,019 models became the Human UCB Ig-seq models dataset. The resulting total CDR length distribution of this set of models also proved to be similar to that of the set of 137 CSTs (Fig. S4B).

Antibody structures and models. Models of all antibodies were generated using the latest implementation of ABodyBuilder (11), used as published, except for the inclusion of PEARS (15) to model side chains. For all antibodies, ABodyBuilder was prevented from using sequence-identical templates, to ensure all structures were genuine models. The 31 unbound and 25 bound Fab structures used to estimate therapeutic model accuracy were X-ray crystal structures deposited in the PDB (16) (before May 4th, 2018). Bound structures were used only if no unbound equivalent existed. If multiple unbound structures were available, the lowest resolution crystal was selected. If multiple bound structures were available, the crystals containing the native antigen were prioritized over those containing synthetic constructs, and then the lowest resolution crystal was chosen. Accuracy was measured using an in-house backbone RMSD calculator, with framework RMSDs obtained after aligning all Fv C_α atoms, and CDR RMSDs calculated after aligning only framework C_α atoms - a standard protocol in the field (17). Case study proprietary sequences were modeled on-site at MedImmune for IP reasons. All MedImmune validation mAbs were models, despite ABodyBuilder having access to additional in-house structural information and their setup permitting sequence identical templates. The web version of TAP does not permit sequence-identical templates in the ABodyBuilder modeling protocol.

Statistical Sampling for the TAP Metrics. We performed statistical sampling over all metric distributions to add error bars to the threshold values. Across 1000 repeats, we randomly selected 200 of the 242 CSTs, and calculated their amber and red threshold values. The results are presented in Table S4, and show that our threshold values are relatively robust to selecting different therapeutics.

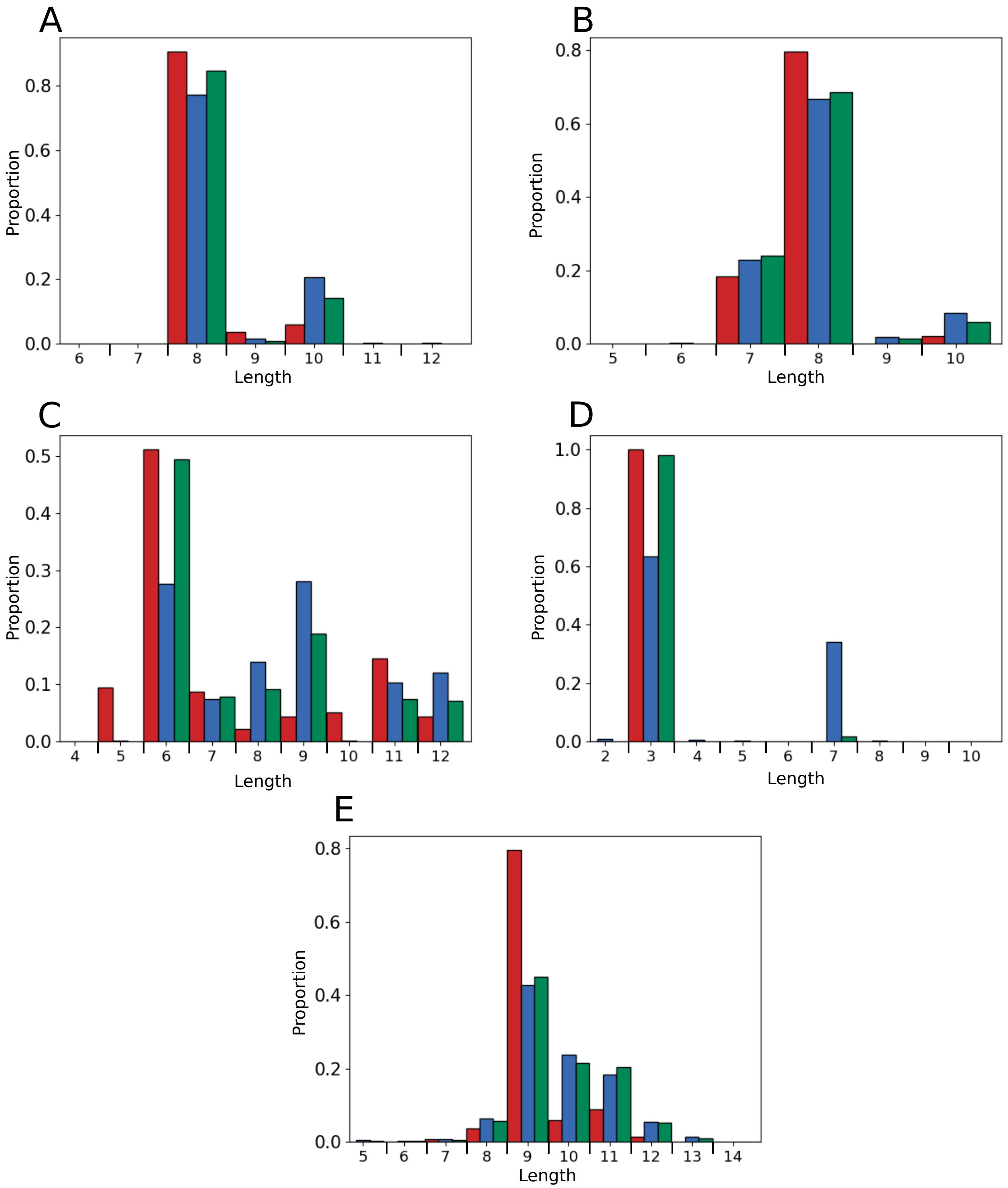

Fig. S1. Comparing the (*A*) CDRH1, (*B*) CDRH2, (*C*) CDRL1, (*D*) CDRL2, and (*E*) CDRL3 length distributions of the 137 CST dataset (red), human VdH Ig-seq non-redundant CDRs (blue), and human VdH Ig-seq non-redundant chains (green). The VdH Ig-seq dataset contains 551,193 non-redundant heavy chains, 1,359,745 non-redundant light chains, and the following numbers of non-redundant CDR sequences: 86,345 CDRH1s, 39,449 CDRH2s, 105,458 CDRH3s, 107,721 CDRL1s, 5,276 CDRL2s, and 235,372 CDRL3s. Lengths which occur very rarely on the scale of non-redundant chains can appear much more often on the scale of non-redundant CDRs (e.g. CDRL2). This is because there are far fewer non-redundant CDR sequences for each CDR type than there are non-redundant chains, and non-redundant chains can contain the same CDR sequence (so frequently expressed CDRs of a certain length dominate the distribution).

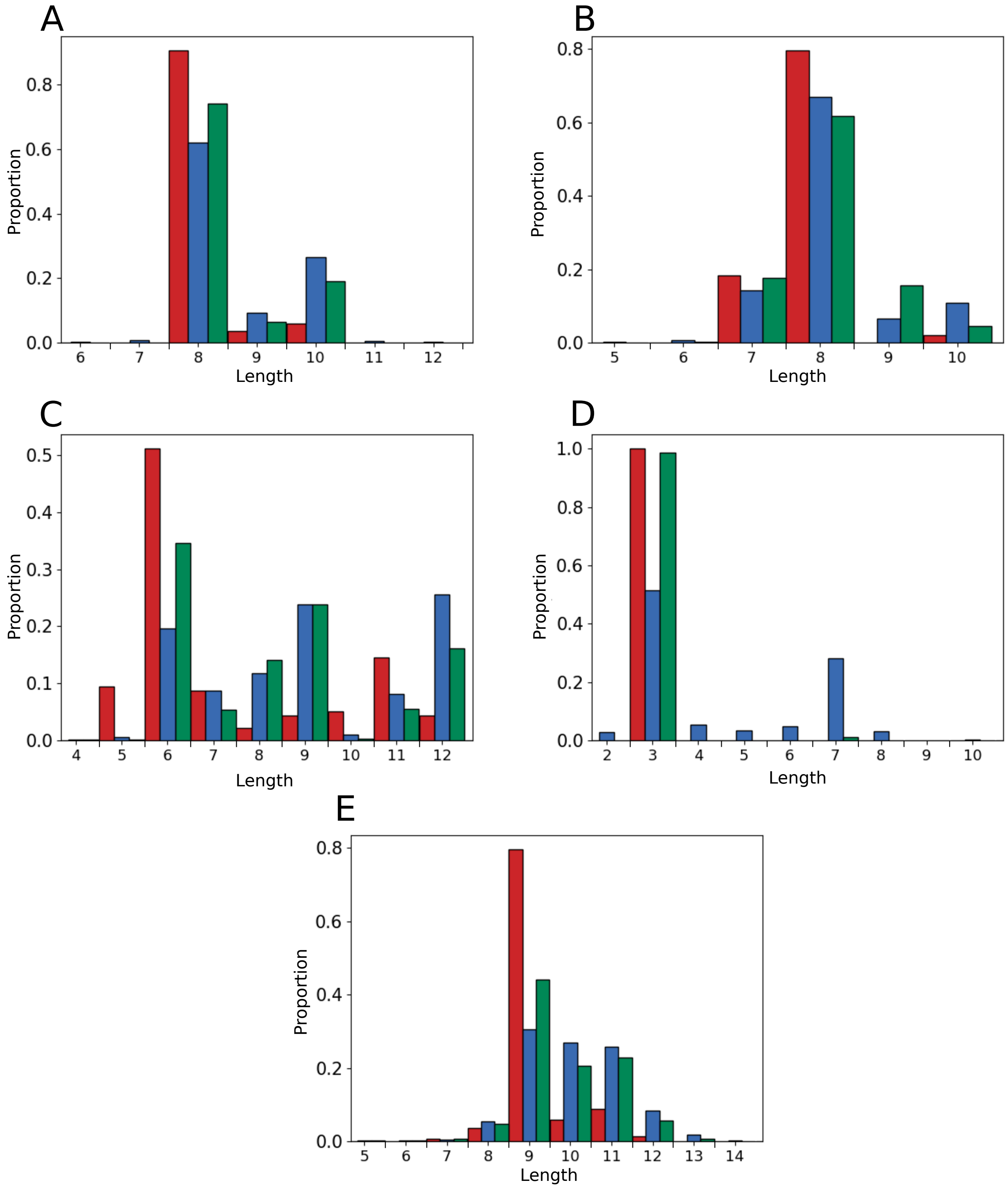

Fig. S2. Comparing the (*A*) CDRH1, (*B*) CDRH2, (*C*) CDRL1, (*D*) CDRL2, and (*E*) CDRL3 length distributions of the 137 CST dataset (red), human UCB Ig-seq non-redundant CDRs (blue), and human UCB Ig-seq non-redundant chains (green). The UCB Ig-seq dataset contains 4,587,907 non-redundant heavy chains, 7,120,100 non-redundant light chains, and the following numbers of non-redundant CDR sequences: 174,490 CDRH1s, 279,873 CDRH2s, 1,696,918 CDRH3s, 455,125 CDRL1s, 8,708 CDRL2s, and 980,158 CDRL3s.

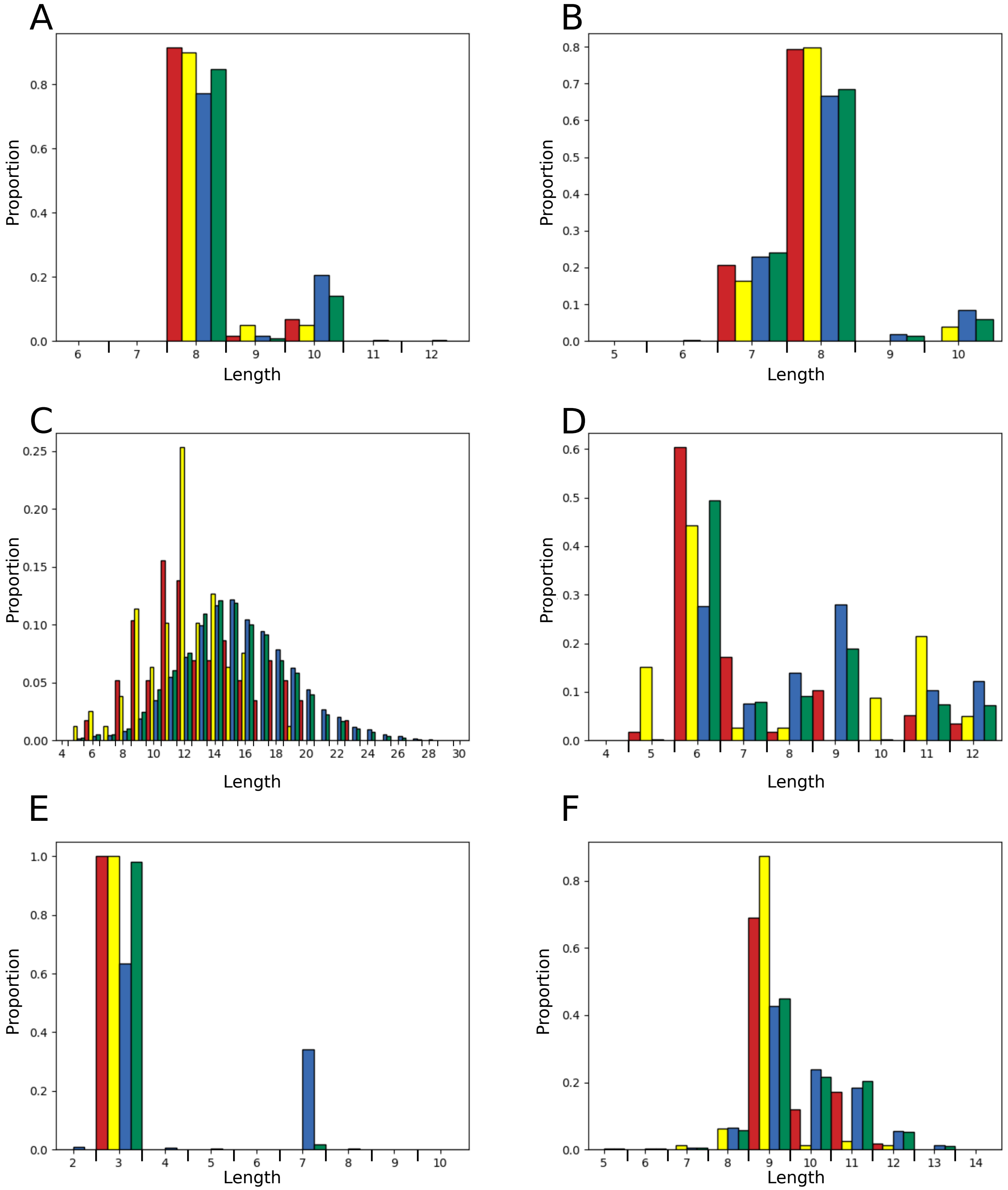

Fig. S3. The (*A*) CDRH1, (*B*) CDRH2, (*C*) CDRH3, (*D*) CDRL1, (*E*) CDRL2, and (*F*) CDRL3 length distributions of the 58 human CSTs (red), 79 humanized, chimeric, or mouse CSTs (yellow), human VdH Ig-seq non-redundant CDRs (blue), and human VdH Ig-seq non-redundant chains (green).

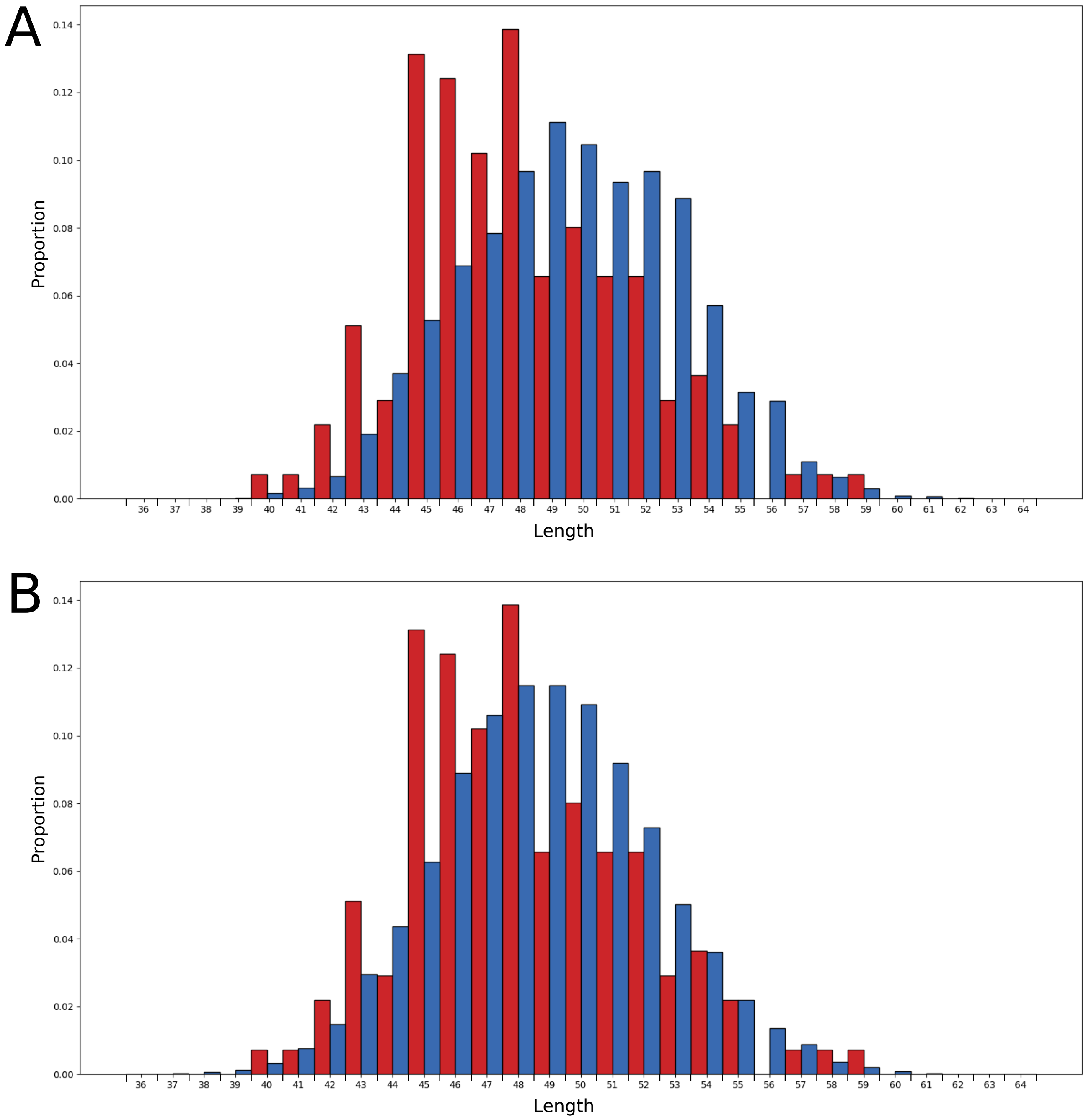

Fig. S4. The total CDR length distributions for (A) the 137 CST (red) and the human VdH Ig-seq models (blue), and for (B) the 137 CST (red) and the human UCB Ig-seq models (blue).

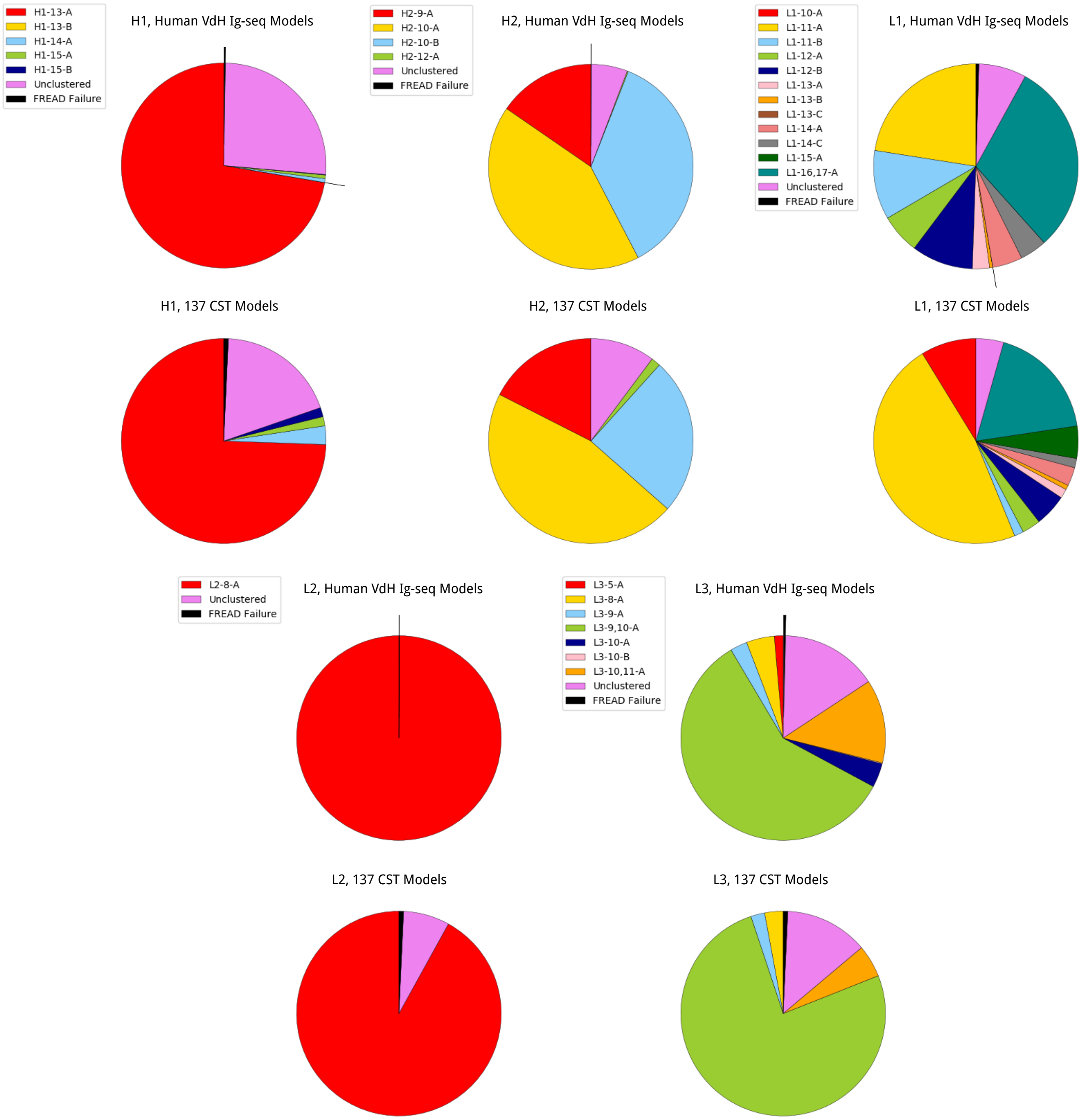

Fig. S5. Length-independent canonical form assignments for the 137 CST and human VdH Ig-seq models. Canonical form colors are consistent with Figure S6.

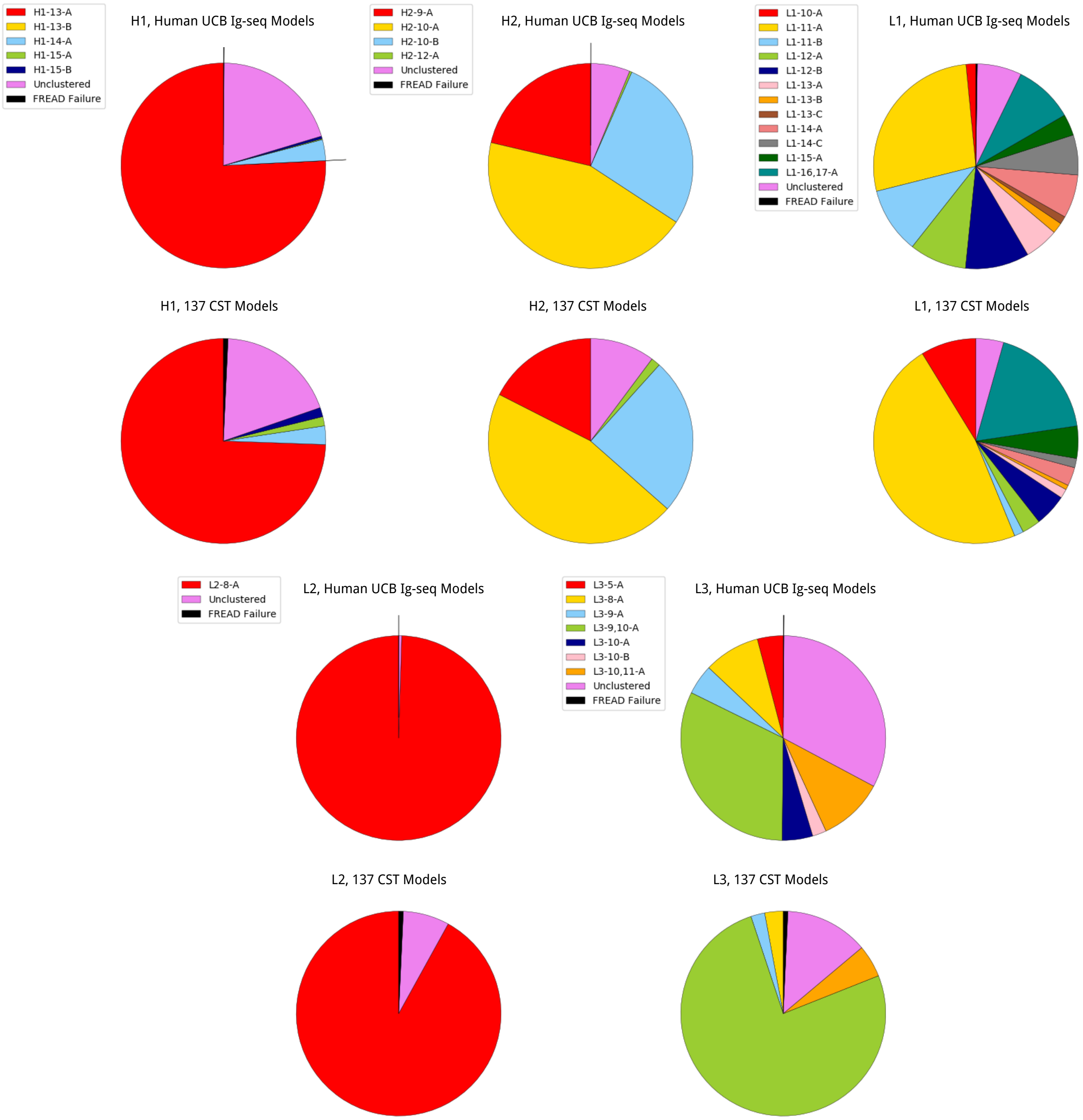

Fig. S6. Length-independent canonical form assignments for the 137 CST and human UCB Ig-seq models.

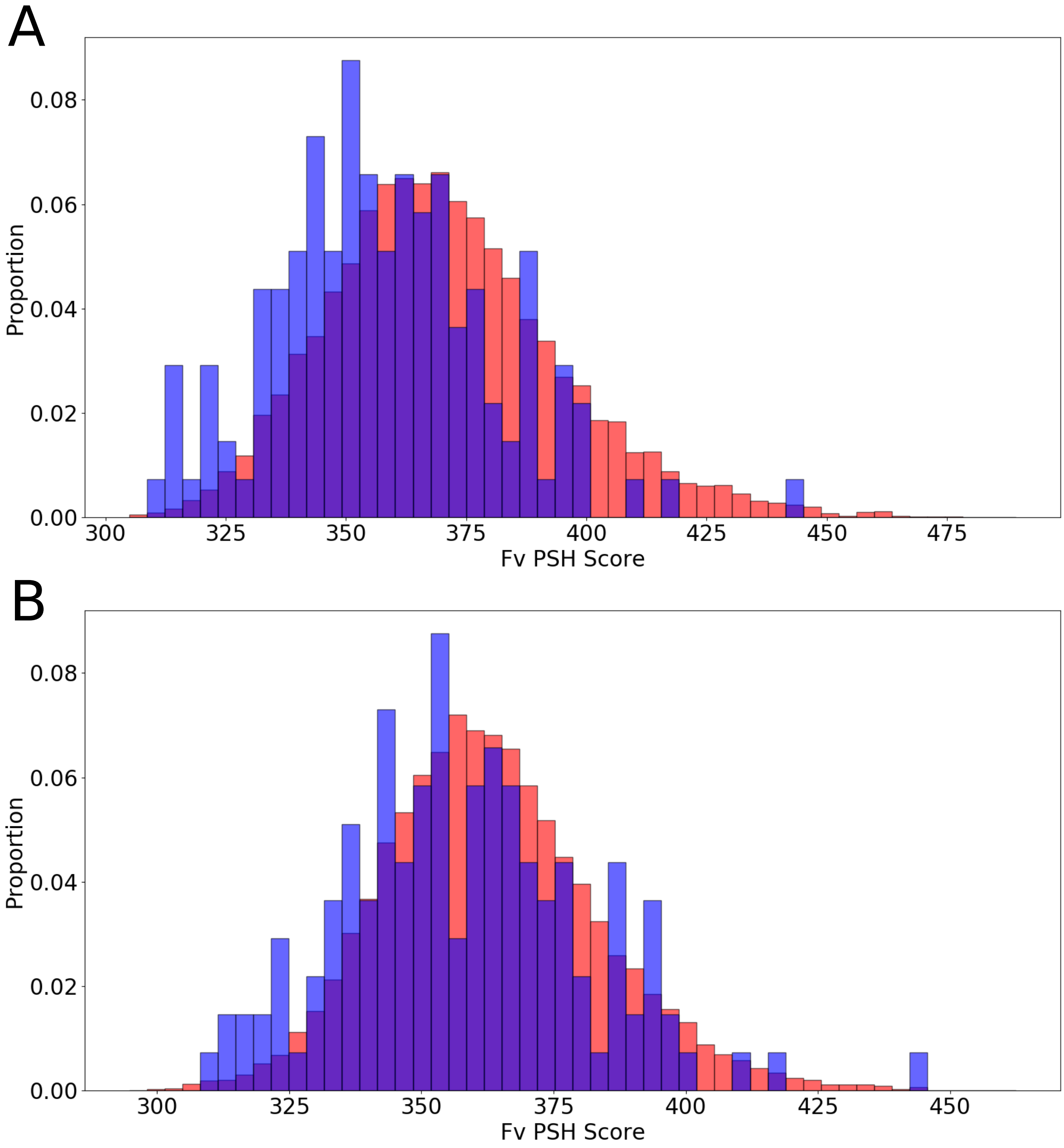

Fig. S7. Fv Region PSH scores (A) across the 137 CST (blue) and human VdH Ig-seq (red) model datasets, and (B) across the 137 CST (blue) and human UCB Ig-seq (red) model datasets (Kyte & Doolittle hydrophobicity scale (18)). The mean value for the human VdH Ig-seq model dataset was 370.56 $\pm$ 24.45, while for the human UCB Ig-seq model dataset was 363.13 $\pm$ 20.64.

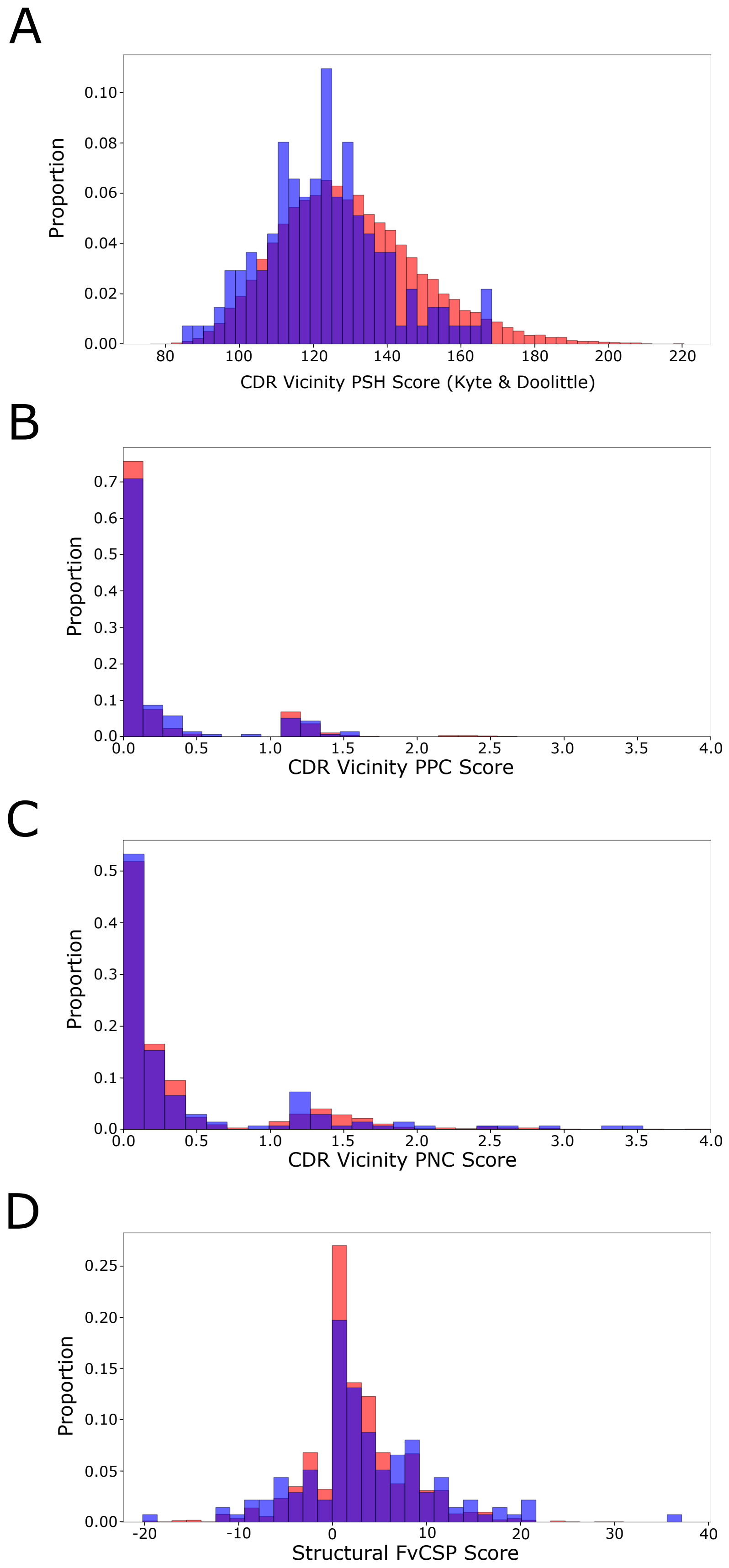

Fig. S8. Distributions for the 137 CSTs (red) and the 19,019 human UCB Ig-seq models (blue) for the (A) CDR Vicinity PSH, (B) CDR Vicinity PPC, (C) CDR Vicinity PNC, and (D) SFvCSP metrics. The mean values for the human UCB Ig-seq models (for comparison with Table S2) are 130.10 $\pm$ 19.53, 0.18 $\pm$ 0.41, 0.36 $\pm$ 0.63, and 2.52 $\pm$ 5.54 respectively.

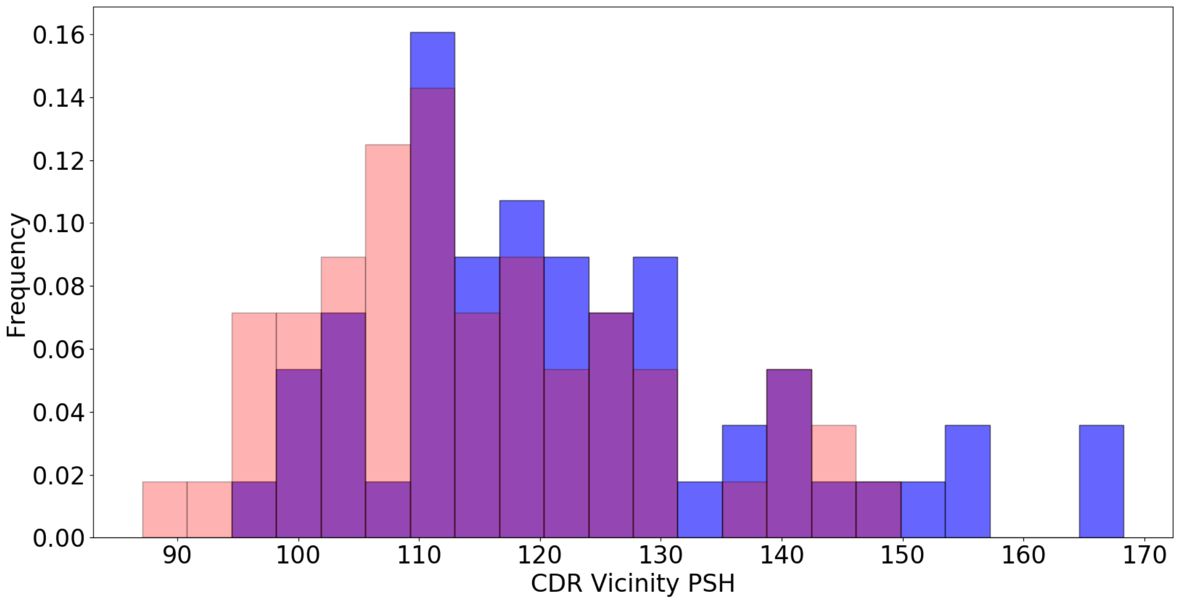

Fig. S9. A comparison between CDR Vicinity PSH scores for 56 CST crystal structures (red) and their models (blue). Models tend to result in higher PSH scores (a mean bias of +7.96) than structures.

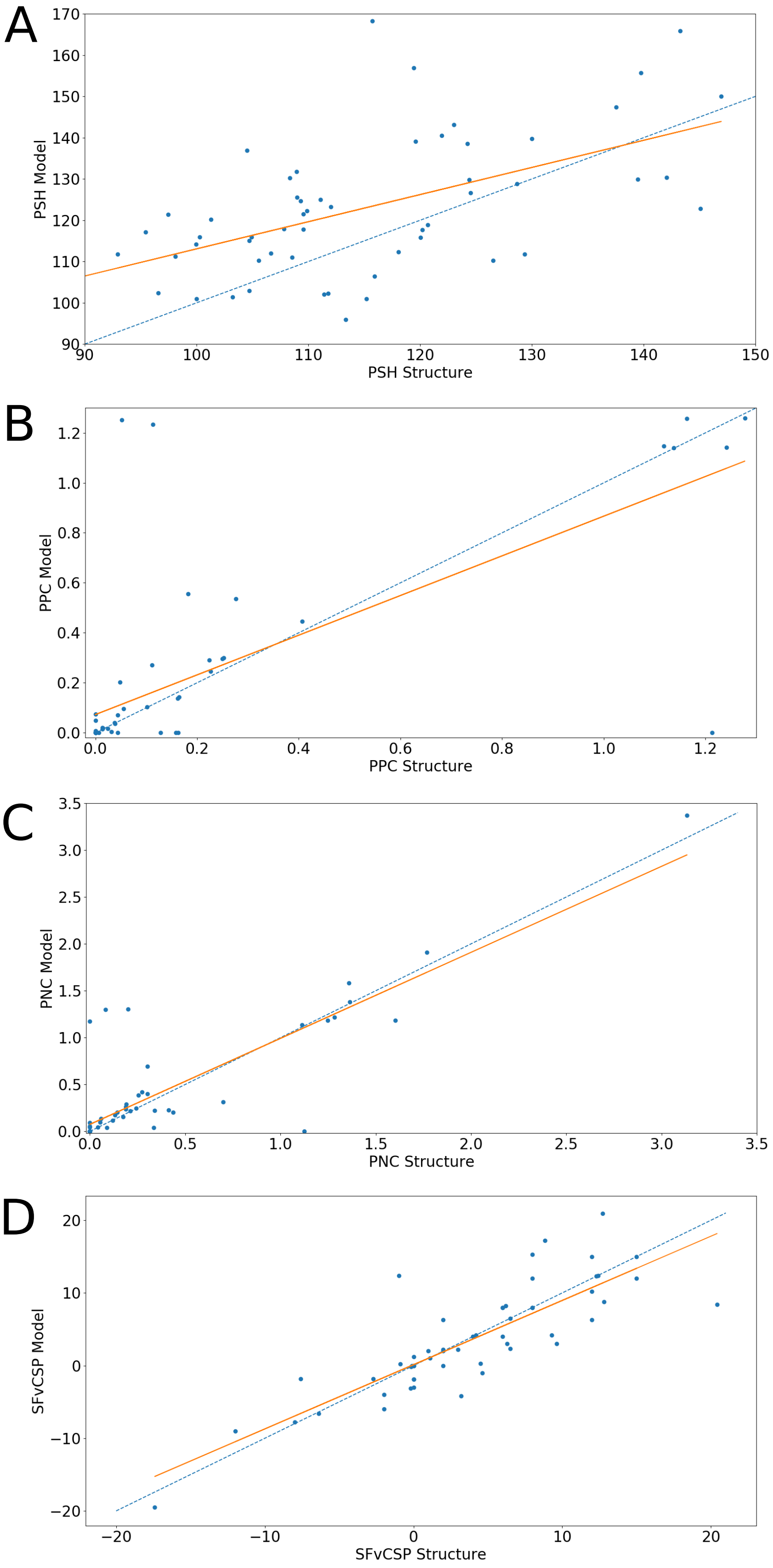

Fig. S10. Plots of therapeutic crystal structure values (x-axis) against therapeutic model values (y-axis) for the CDR Vicinity (A) PSH, (B) PPC, (C) PNC metrics and the (D) SFvCSP metric. The identity line (blue dashes) and a line of best fit is plotted for each metric. Pearson correlation coefficients and p-values are as follows. PSH: ρ = 0.558, p = 7.98 x 10^{-6}; PPC: ρ = 0.723, p = 3.04 x 10^{-10}; PNC: ρ = 0.858, p = 2.78 x 10^{-17}; SFvCSP: ρ = 0.835, p = 1.27 x 10^{-15}.

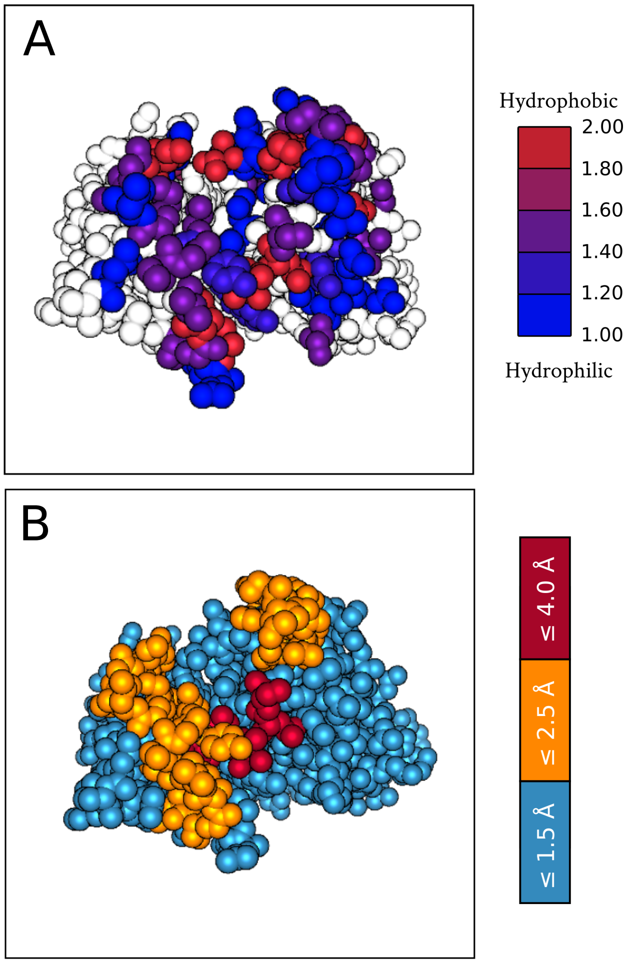

Fig. S11. (A) An example TAP web application output showing the heavy atoms of an antibody as spheres colored by the hydrophobicity (Kyte & Doolittle scale, normalized between 1 and 2) of each residue in the CDR vicinity. (B) The ABodyBuilder predicted model accuracy assignments (11) for each IMGT region, with heavy atoms shown as spheres. These are colored according to three backbone RMSD thresholds at a 75% confidence interval (both thresholds and confidence intervals can be modified in the web application). Better quality models will yield more reliable TAP metric values.

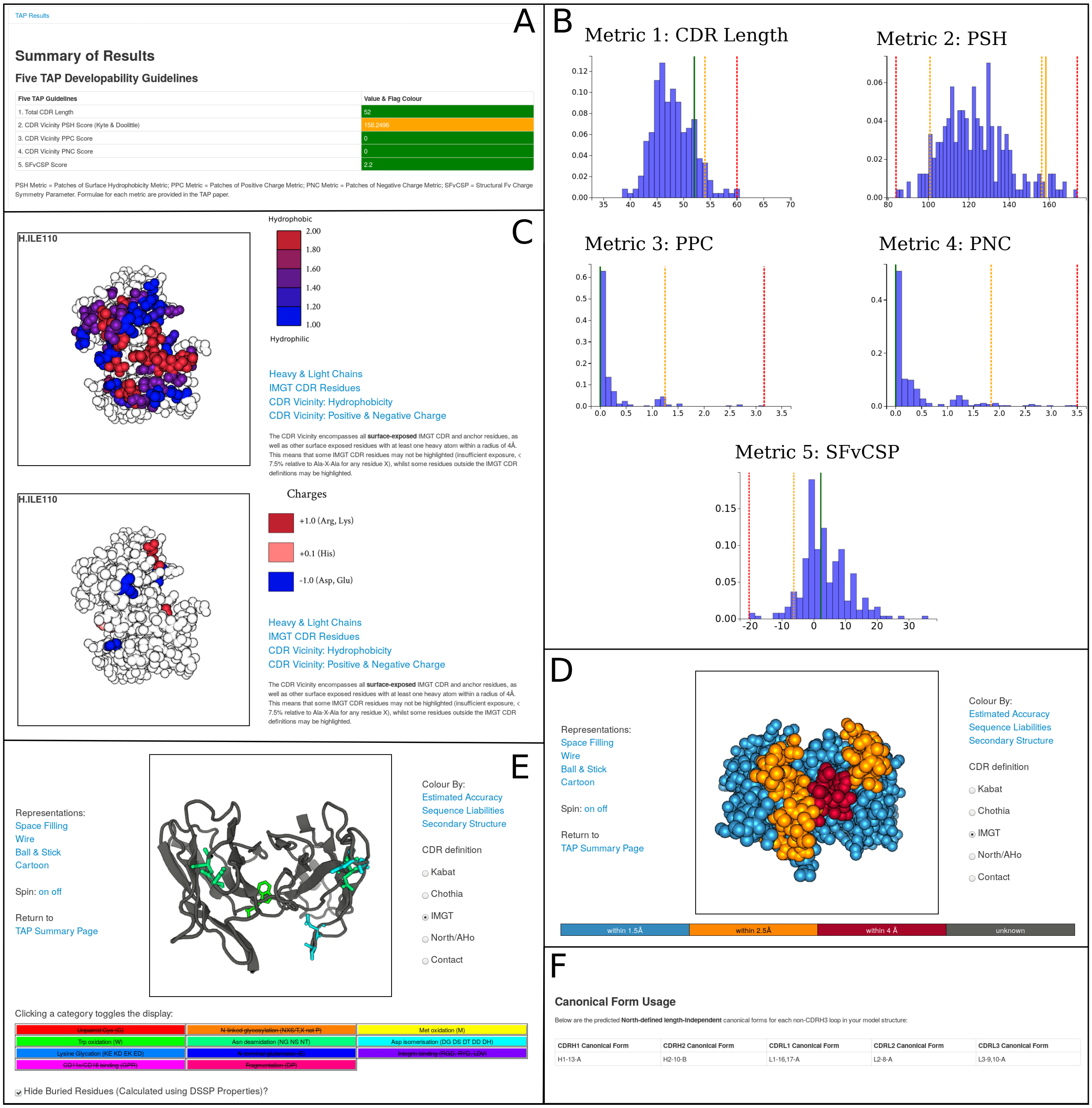

Fig. S12. A sample TAP web application output. (A) The five TAP metric values are reported in a table, whose cells are colored by assigned flag. (B) The metric values are also shown against the distributions of the 242 CSTs. Amber and red dashed lines indicate the amber and red flag threshold values, and the solid lines the value for the inputted antibody, colored by assigned flag. (C) A molecular viewer allows for visualization of hydrophobicity and charge in the CDR vicinity. In this antibody, the charge is evenly spread across the CDR vicinity, while there is a large patch of hydrophobicity spanning the CDRH1 and CDRH3 loops. (D) Predicted model accuracy can be visualized across each IMGT region. The confidence interval and all RMSD cutoffs can be altered. (E) Potential sequence liabilities are shown on a cartoon representation of the antibody. A check box is available to hide buried residues. (F) North-defined, length-independent canonical forms are reported for each CDR loop in the antibody.

Table S1. Backbone Root-Mean-Square Deviation across each region for the 56 of 137 CSTs with unbound/bound PDB reference structures. All models were made with ABodyBuilder, without using sequence identical templates. Gaps are assigned if the PDB structure has missing residues, precluding an accurate RMSD assignment.

Therapeutic	Unbound/Bound (PDB Code)	Framework (Å)	CDRH1 (Å)	CDRH2 (Å)	CDRH3 (Å)	CDRL1 (Å)	CDRL2 (Å)	CDRL3 (Å)
1. Abituzumab	Bound (4O02)	1.357	1.101	1.494	2.516	0.919	1.307	1.143
2. Adalimumab	Unbound (4NYL)	0.711	0.646	0.539	2.871	0.922	0.435	0.932
3. Alemtuzumab	Unbound (1BEY)	0.701	1.766	1.143	3.086	0.822	1.011	1.122
4. Anifrolumab	Unbound (4QXG)	0.575	0.674	0.864	1.492	2.305	0.746	1.275
5. Atezolizumab	Bound (5XXY)	0.734	1.146	1.785	3.403	1.481	0.552	1.451
6. Bapineuzumab	Bound (4OJF)	1.357	1.101	1.494	2.516	0.919	1.307	1.143
7. Basiliximab	Unbound (1MIM)	0.761	0.71	0.739	1.067	0.593	0.428	1.127
8. Belimumab	Unbound (5Y9K)	0.714	3.499	1.012	3.940	4.354	0.809	2.451
9. Bimagrumab	Unbound (5NHW)	0.819	1.842	0.858	0.553	1.027	0.606	4.669
10. Briakinumab	Unbound (5N2K)	0.756	0.752	0.529	2.645	1.655	0.686	5.077
11. Canakinumab	Unbound (4G5Z)	0.824	0.789	0.784	2.001	1.296	0.712	0.990
12. Carlumab	Unbound (4DN3)	0.692	2.816	2.702	1.780	1.707	0.416	1.640
13. Certolizumab	Unbound (5WUV)	0.578	1.215	1.253	3.648	0.412	0.195	2.362
14. Cetuximab	Unbound (1YY8)	0.810	0.251	0.302	0.348	0.236	0.139	0.401
15. Crenezumab	Unbound (5KMV)	0.658	0.829	0.618	0.733	1.127	0.579	0.405
16. Daclizumab	Unbound (3NFS)	0.682	0.672	0.925	1.310	1.267	0.683	0.849
17. Drozitumab	Bound (4OD2)	0.840	0.530	1.134	4.999	2.073	2.118	2.265
18. Eculizumab	Bound (5I5K)	1.752	1.028	1.077	2.006	1.114	0.589	1.345
19. Efalizumab	Unbound (3EO9)	0.740	1.049	2.069	2.038	0.617	0.278	1.123
20. Epratuzumab	Unbound (5VKK)	-	-	2.260	-	0.995	0.496	1.651
21. Fresolimumab	Unbound (3EO0)	0.652	3.679	1.193	8.882	1.829	0.173	0.729
22. Gantenerumab	Bound (5CSZ)	0.535	0.679	2.712	3.197	2.165	0.418	1.112
23. Gevokizumab	Unbound (4G6K)	1.014	2.592	1.219	1.332	0.917	0.447	0.932
24. Guselkumab	Unbound (4M6N)	1.290	0.431	0.485	3.180	1.371	0.518	1.490
25. Ibalizumab	Bound (3O2D)	1.132	0.828	1.627	3.933	0.957	0.409	0.920
26. Infliximab	Unbound (5VH3)	0.640	1.049	1.379	2.732	0.636	0.176	0.330
27. Ipilimumab	Bound (5TRU)	0.568	0.649	0.816	2.099	0.865	0.279	0.870
28. Lampalizumab	Bound (4D9Q)	1.008	0.387	0.549	3.792	0.547	0.662	0.873
29. Lebrikizumab	Bound (4I77)	0.503	1.575	1.663	2.006	0.645	0.464	1.051
30. Matuzumab	Unbound (3C08)	1.287	-	1.255	9.966	-	0.547	2.780
31. Motavizumab	Bound (3QWO)	1.231	3.299	1.684	3.827	1.949	0.504	3.314
32. Muromonab	Bound (1SY6)	0.193	0.441	0.553	2.101	0.469	0.333	1.119
33. Natalizumab	Bound (4IRZ)	0.838	3.180	0.874	4.652	1.161	0.512	1.476
34. Necitumumab	Bound (3B2U)	0.696	1.472	1.372	5.869	0.648	0.733	0.744
35. Nimotuzumab	Unbound (3GKW)	1.846	4.046	1.737	7.573	1.049	0.424	1.291
36. Nivolumab	Unbound (5GGQ)	0.847	0.597	0.851	2.393	3.280	0.390	0.851
37. Obinutuzumab	Unbound (3PP3)	0.792	0.746	0.368	5.147	2.972	0.726	0.804
38. Ofatumumab	Unbound (3GIZ)	0.909	0.584	2.226	3.581	0.410	0.292	1.119
39. Olokizumab	Bound (4CNI)	1.185	0.518	0.756	1.271	0.348	0.523	0.898
40. Omalizumab	Unbound (4X7S)	1.180	1.760	0.896	3.177	0.807	0.341	1.287
41. Onartuzumab	Bound (4K3J)	1.228	1.655	1.732	3.016	0.937	0.410	1.028
42. Palivizumab	Unbound (2HWZ)	0.645	0.766	0.392	1.573	0.672	0.528	2.111
43. Panitumumab	Bound (5SX4)	0.910	1.312	1.567	1.645	0.273	0.781	0.506
44. Pembrolizumab	Unbound (5DK3)	0.755	0.424	0.466	3.536	1.175	0.553	1.099
45. Pertuzumab	Bound (5JXE)	0.970	3.007	1.696	2.920	2.053	0.383	0.985
46. Pinatuzumab	Bound (6AND)	0.909	1.643	3.983	3.554	2.754	0.422	0.441
47. Ponezumab	Bound (3U0T)	1.438	1.837	0.753	4.425	1.882	0.339	0.758
48. Ramucirumab	Unbound (3S34)	0.718	0.419	0.962	0.910	0.370	0.271	0.576
49. Ranibizumab	Bound (1CZ8)	0.559	1.085	1.245	4.065	0.546	0.702	0.936
50. Rituximab	Unbound (4KAQ)	0.942	0.359	0.804	4.815	0.477	0.373	0.693
51. Sifalimumab	Bound (4YPG)	0.790	0.782	0.633	3.819	3.670	1.055	2.911
52. Tanezumab	Bound (4EDW)	0.919	1.828	0.898	2.065	0.352	0.792	0.594
53. Tralokinumab	Bound (5L6Y)	0.403	0.698	0.630	4.073	1.054	0.271	2.526
54. Trastuzumab	Bound (4HKZ)	0.595	0.449	0.814	3.226	0.278	0.192	0.537
55. Tremelimumab	Unbound (5GGU)	0.696	0.526	0.700	4.912	0.525	0.214	0.957
56. Ustekinumab	Unbound (3HMW)	0.495	0.411	1.054	2.363	0.254	0.254	0.592
MEAN VALUES		0.831	1.181	1.176	3.093	1.144	0.517	1.288

Table S2. Average TAP Metric Values for the 242 CST models, 14,072 Human VdH Ig-seq models, 56 CST crystal structures, and 33 Human non-engineered (NE), non-redundant crystal structures. Results are reported as mean values $\pm$ one standard deviation. The identities of the 33 human, non-redundant, non-engineered structures are listed in Dataset S1, and the 56 therapeutic structures are listed in Table S1.

Metric	242 CST Models	14,072 VdH Ig-seq Models	56 CST Structures	33 NE Human Structures
Total CDR Length (L)	48.02 $\pm$ 3.77	49.75 $\pm$ 3.49	47.64 $\pm$ 3.20	51.03 $\pm$ 4.35
PSH, CDR Vicinity (Kyte)	123.30 $\pm$ 16.60	133.76 $\pm$ 21.08	114.92 $\pm$ 14.00	124.61 $\pm$ 16.54
PPC, CDR Vicinity	0.24 $\pm$ 0.49	0.25 $\pm$ 0.52	0.19 $\pm$ 0.36	0.44 $\pm$ 0.73
PNC, CDR Vicinity	0.41 $\pm$ 0.66	0.38 $\pm$ 0.62	0.35 $\pm$ 0.60	0.70 $\pm$ 1.09
SFvCSP	3.34 $\pm$ 7.44	3.67 $\pm$ 7.40	3.81 $\pm$ 6.87	3.44 $\pm$ 7.56

Table S3. The numbers of a test set of 105 CSTs that were assigned amber or red flags across the five TAP guideline metrics (flagging thresholds set by the 137 CST dataset).

Metric	137 CST Amber Flag Region	Number Amber Flagged	137 CST Red Flag Region	Number Red Flagged
Total CDR Length (L)	$54 \leq L \leq 59$	6	$L > 59$	2*
PSH, CDR Vicinity (Kyte)	$85.65 \leq PSH \leq 98.74$	2	$PSH < 85.65$	1
	$155.76 \leq PSH \leq 171.91$	5	$PSH > 171.91$	1*
PPC, CDR Vicinity	$1.23 \leq PPC \leq 1.51$	1	PPC > 1.51	5*
PNC, CDR Vicinity	$1.90 \leq PNC \leq 3.50$	4	PNC > 3.50	0
SFvCSP	$-19.50 \leq SFvCSP \leq -9.00$	1	SFvCSP < -19.50	1

*Erenumab flagged for each of these properties.

Table S4. Statistical Sampling of the TAP Metrics. Results are presented as the mean value $\pm$ one standard deviation, calculated over 1,000 repeats of randomly sampling 200 CSTs.

Metric	Amber Flag Threshold Value	Red Flag Threshold Value
Total CDR Length (L)	54.13 ± 0.32	59.97 ± 0.21
PSH, CDR Vicinity (Kyte)	99.64 ± 0.76	84.28 ± 1.20
	156.48 ± 1.57	172.84 ± 2.18
PPC, CDR Vicinity	1.24 ± 0.02	3.07 ± 0.20
PNC, CDR Vicinity	1.83 ± 0.08	3.47 ± 0.10
SFvCSP	-6.49 ± 0.56	-20.19 ± 0.65

Table S5. TAP values across kappa and lambda models.

Dataset	TAP Metric	Kappa Subset ($\mu \pm \sigma$)	Lambda Subset ($\mu \pm \sigma$)
242 CST Models	PSH	120.89 $\pm$ 15.10	142.03 $\pm$ 19.09
	PPC	0.21 $\pm$ 0.47	0.53 $\pm$ 0.56
	PNC	0.38 $\pm$ 0.64	0.60 $\pm$ 0.77
	SFvCSP	3.82 $\pm$ 7.38	1.67 $\pm$ 7.87
14,072 VdH Ig-seq Models	PSH	131.27 $\pm$ 21.41	141.68 $\pm$ 17.82
	PPC	0.17 $\pm$ 0.40	0.52 $\pm$ 0.73
	PNC	0.27 $\pm$ 0.48	0.74 $\pm$ 0.83
	SFvCSP	4.56 $\pm$ 7.44	0.84 $\pm$ 6.48
19,019 UCB Ig-seq Models	PSH	125.40 $\pm$ 18.56	139.66 $\pm$ 17.88
	PPC	0.11 $\pm$ 0.31	0.31 $\pm$ 0.53
	PNC	0.22 $\pm$ 0.40	0.65 $\pm$ 0.88
	SFvCSP	3.67 $\pm$ 5.30	0.12 $\pm$ 5.24

Table S6. 242 CST TAP values split by clinical development.

TAP Metric	117 Phase II ($\mu \pm \sigma$)	55 Phase III ($\mu \pm \sigma$)	69 Approved/Pre-registration ($\mu \pm \sigma$)
Total CDR Length	48.12 $\pm$ 3.90	47.55 $\pm$ 3.30	48.23 $\pm$ 3.86
PSH	122.82 $\pm$ 17.08	122.60 $\pm$ 15.50	123.88 $\pm$ 17.40
PPC	0.24 $\pm$ 0.51	0.26 $\pm$ 0.55	0.24 $\pm$ 0.40
PNC	0.38 $\pm$ 0.61	0.58 $\pm$ 0.81	0.30 $\pm$ 0.58
SFvCSP	3.03 $\pm$ 7.00	4.59 $\pm$ 8.74	3.75 $\pm$ 7.03

Table S7. 242 CST TAP values split by drug campaign status. (NB: The status of the missing five CSTs is unknown).

TAP Metric	178 Active/Approved ($\mu \pm \sigma$)	59 Discontinued ($\mu \pm \sigma$)
Total CDR Length	47.83 $\pm$ 3.59	48.64 $\pm$ 4.32
PSH	122.65 $\pm$ 16.57	124.19 $\pm$ 17.81
PPC	0.25 $\pm$ 0.52	0.20 $\pm$ 0.36
PNC	0.44 $\pm$ 0.71	0.31 $\pm$ 0.47
SFvCSP	3.05 $\pm$ 7.20	5.12 $\pm$ 7.84

Table S8. 242 CST TAP values split by species origin.

TAP Metric	101 Human ($\mu \pm \sigma$)	108 Humanized ($\mu \pm \sigma$)	30 Chimeric ($\mu \pm \sigma$)	3 Mouse ($\mu \pm \sigma$)
Total CDR Length	48.68 $\pm$ 4.09	47.80 $\pm$ 3.42	46.77 $\pm$ 3.55	46.33 $\pm$ 1.25
PSH	127.76 $\pm$ 18.56	120.90 $\pm$ 14.20	115.73 $\pm$ 15.58	117.26 $\pm$ 9.44
PPC	0.29 $\pm$ 0.58	0.20 $\pm$ 0.36	0.26 $\pm$ 0.55	0.05 $\pm$ 0.06
PNC	0.34 $\pm$ 0.56	0.50 $\pm$ 0.75	0.30 $\pm$ 0.63	0.50 $\pm$ 0.50
SFvCSP	4.06 $\pm$ 7.44	3.13 $\pm$ 7.80	3.29 $\pm$ 5.99	7.58 $\pm$ 6.75

Additional data table S1 (33_Human_Noneng_Nonred_PDBs.xlsx)
A spreadsheet of the 33 human, non-engineered, non-redundant PDB codes for which we calculated TAP metric values.

Additional data table S2 (242_Clinical_Stage_Therapeutics.xlsx)
A spreadsheet of all 242 CSTs referenced in this study. It includes their variable domain sequences, furthest progression through clinical trials, and whether or not the drug is still in active development. The table is colored to show which CSTs belong to the 137 CST and 105 CST datasets.

Additional data table S3 (242_CST_Metric_Values.xlsx)
A spreadsheet of the TAP metric values for all 242 CSTs.

The 242 CST and 14,072 human VdH Ig-seq models are available for download at: http://opig.stats.ox.ac.uk/resources.

References

1. Vander Heiden JA, et al. (2017) Dysregulation of b cell repertoire formation in myasthenia gravis patients revealed through deep sequencing. *J. Immunol.* 198:1460–1473.
2. Ye J, Ma N, Madden TL, Ostell JM (2013) IgBLAST: an immunoglobulin variable domain sequence analysis tool. *Nucleic Acids Res* 41:34–40.
3. Dunbar J, Deane CM (2015) ANARCI: Antigen receptor numbering and receptor classification. *Bioinformatics* 32(2):298–300.
4. Kovaltsuk A, et al. (2018) Observed antibody space: A resource for data mining next-generation sequencing of antibody repertoires. *J. Immunol.* 201(8):2502–2509.
5. Al-Lazikani B, Lesk AM, Chothia C (1997) Standard conformations for the canonical structures of immunoglobulins. *J Mol Biol* 273(4):927–948.
6. Wong WK, Leem J, Lewis AP, , et al. (2018) Scalop: Sequence-based antibody canonical loop structure prediction.
7. Deane CM, Blundell TL (2001) CODA: a combined algorithm for predicting the structurally variable regions of protein models. *Protein Sci* 10(3):599–612.
8. Choi Y, Deane CM (2010) FREAD revisited: Accurate loop structure prediction using a database search algorithm. *Proteins* 78(6):1431–1440.
9. Choi Y, Deane CM (2011) Predicting antibody complementarity determining region structures without classification. *Mol BioSyst* 7(12):3327–3334.
10. Dunbar J, et al. (2014) SAbDab: The structural antibody database. *Nucleic Acids Res* 42(D1):1140–1146.
11. Leem J, Dunbar J, Georges G, Shi J, Deane CM (2016) ABodyBuilder: Automated antibody structure prediction with data–driven accuracy estimation. *mAbs* 8(7):1259–1268.
12. Nowak J, et al. (2016) Length-independent structural similarities enrich the antibody CDR canonical class model. *mAbs* 8(4):751–760.
13. Kabsch W (1976) A solution for the best rotation to relate two sets of vectors. *Acta Crystallographica A* 32(5):922–923.
14. Sokal RR, Michener CD (1958) A Statistical Method for Evaluating Systematic Relationships. *Univ Kansas Sci Bull* 38:1409–1438.
15. Leem J, Georges G, Shi J, Deane CM (2018) Antibody side chain conformations are position-dependent. *Proteins* 86(4):383–392.
16. Berman HM, et al. (2000) The protein data bank. *Nucleic Acids Res* 28(1):235–242.
17. Almagro JC, et al. (2014) Second antibody modeling assessment (AMA-II). *Proteins* 82(8):1553–1562.
18. Kyte J, Doolittle RF (1982) A simple method for displaying the hydropathic character of a protein. *J Mol Biol* 157(1):105–132.
